# TRIM21‐Promoted FSP1 Plasma Membrane Translocation Confers Ferroptosis Resistance in Human Cancers

**DOI:** 10.1002/advs.202302318

**Published:** 2023-08-16

**Authors:** Jun Gong, Yuhui Liu, Wenjia Wang, Ruizhi He, Qilong Xia, Lin Chen, Chunle Zhao, Yang Gao, Yongkang Shi, Yu Bai, Yangwei Liao, Qi Zhang, Feng Zhu, Min Wang, Xu Li, Renyi Qin

**Affiliations:** ^1^ Department of Biliary‐Pancreatic Surgery Affiliated Tongji Hospital Tongji Medical College Huazhong University of Science and Technology 1095 Jiefang Ave Wuhan Hubei 430030 China; ^2^ Hubei Key Laboratory of Hepato‐Pancreato‐Biliary Diseases Affiliated Tongji Hospital Tongji Medical College Huazhong University of Science and Technology 1095 Jiefang Ave Wuhan Hubei 430030 China; ^3^ Institute of Integrated Traditional Chinese and Western Medicine Affiliated Tongji Hospital Tongji Medical College Huazhong University of Science and Technology 1095 Jiefang Ave Wuhan Hubei 430030 China; ^4^ Department of Plastic and Cosmetic Surgery Affiliated Tongji Hospital Tongji Medical College Huazhong University of Science and Technology 1095 Jiefang Ave Wuhan Hubei 430030 China

**Keywords:** cancer, ferroptosis, FSP1, TRIM21, ubiquitination

## Abstract

Ferroptosis, an iron‐dependent form of regulated cell death driven by excessive accumulation of lipid peroxides, has become a promising strategy in cancer treatment. Cancer cells exploit antioxidant proteins, including Ferroptosis Suppressor Protein 1 (FSP1), to prevent ferroptosis. In this study, it is found that the E3 ubiquitin ligase TRIM21 bound to FSP1 and mediated its ubiquitination on K322 and K366 residues via K63 linkage, which is essential for its membrane translocation and ferroptosis suppression ability. It is further verified the protective role of the TRIM21‐FSP1 axis in RSL3‐induced ferroptosis in cancer cells and a subcutaneous tumor model. Moreover, TRIM21 is highly expressed in multiple gastrointestinal (GI) tumors, and its expression is further stimulated upon ferroptosis induction in cancer cells and the KPC mouse model. In summary, This study identifies TRIM21 as a negative regulator of ferroptosis through K63 ubiquitination of FSP1, which can serve as a therapeutic target to enhance the chemosensitivity of tumors based on ferroptosis induction.

## Graphical abstract

FSP1 functions as ferroptosis suppressor parallel to GPx4. This study shows that ferroptosis induction promotes TRIM21 expression, as well as K63 ubiquitination and plasma membrane translocation of FSP1. Furthermore, TRIM21 functions as a negative regulator of ferroptosis by promoting K63 ubiquitination of FSP1. Thus, targeting the TRIM21‐FSP1 axis may provide a novel strategy for promoting ferroptosis sensitivity in cancer therapy.

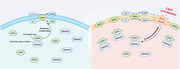

## 1 Introduction

Ferroptosis, a form of regulated cell death discovered by Dixon et al. in 2012,^[^ ^1^ ^]^ is attracting much attention in cancer research. Ferroptosis is characterized by lethal iron‐dependent accumulation of lipid peroxides, which can be prevented by lipophilic antioxidants and iron chelators.^[^ ^2^, ^3^ ^]^ Due to the excessive iron and lipid metabolism combined with the high level of reactive oxygen species (ROS) in cancer cells, ferroptosis has been regarded as a vulnerability in cancer cells and holds great therapeutic potential in cancers, especially in those refractory to conventional therapies.^[^ ^4^, ^5^ ^]^ However, cancer cells have also evolved various defence strategies to counter the accumulated lipid peroxides, including the GPx4‐GSH system,^[^ ^6^ ^]^ DHODH‐CoQH_2_ system,^[^ ^7^ ^]^ GCH1‐BH4 system,^[^ ^8^, ^9^ ^]^ and FSP1‐CoQH_2_/VKH_2_ system.^[^ ^10^, ^11^, ^12^ ^]^ Ferroptosis Suppressor Protein 1 (FSP1), also called AIFM2, was found to be an important ferroptosis suppressor in 2019 by two individual research teams.^[^ ^10^, ^11^ ^]^ FSP1 functions as a NAD(P)H‐dependent oxidoreductase to reduce ubiquinone (CoQ) to ubiquinol (CoQH_2_) or reduce vitamin K to VKH_2_, which acts as a radical‐trapping antioxidant (RTA) in plasma membranes to suppress lipid peroxidation.^[^ ^12^ ^]^ In addition, both teams noted that the ferroptosis suppression ability of FSP1 relies on its plasma membrane localization, which was mediated by N‐myristoylation at glycine‐2. Other researchers have focused on the transcriptional modulation of FSP1. It has been reported that FSP1 is regulated by the KEAP1‐NRF2 pathway at the transcriptional level and that N‐acetyltransferase 10 (NAT10) stabilizes FSP1 mRNA via N4‐acetylation.^[^ ^13^, ^14^ ^]^ However, the posttranslational modifications (PTMs) of FSP1 remain largely undiscovered.

PTMs are covalent modifications that regulate protein structure and functions from protein synthesis to degradation. Phosphorylation, methylation, acetylation, glycosylation, and ubiquitination are critical and prevalent PTMs that occur in most proteins.^[^ ^15^, ^16^ ^]^ Ubiquitination is a reversible process controlled by ubiquitin enzymes and deubiquitinases (DUBs).^[^ ^17^, ^18^ ^]^ Different types of ubiquitination usually have different effects on the substrate.^[^ ^19^, ^20^ ^]^ K48 ubiquitination usually guides the substrate to the proteasome for degradation, which is the most well‐known function of ubiquitination.^[^ ^21^, ^22^ ^]^ However, K63 ubiquitination have diverse effects on the substrate, including affecting protein‒protein interactions,^[^ ^23^, ^24^, ^25^ ^]^ translocating the protein to the nucleus,^[^ ^26^, ^27^ ^]^ vesicle^[^ ^28^, ^29^ ^]^ or plasma membrane,^[^ ^30^, ^31^ ^]^ or targeting the protein to the lysosome for degradation.^[^ ^32^, ^33^ ^]^ With the gradual deepening of research on ubiquitination, the role of K63 ubiquitination has attracted increasing attention.

Tripartite motif (TRIM)‐containing proteins are a family of E3 proteins that contain an N‐terminal RING finger, a B‐box domain, and a coiled‐coil region.^[^ ^34^ ^]^ With more than 80 members, TRIM family proteins are engaged in a wide range of biological processes, including immune regulation, DNA repair, carcinogenesis, and cell death.^[^ ^35^, ^36^ ^]^ Tripartite Motif‐Containing Protein 21 (TRIM21, RNF81, or RO52) is an important member of the TRIM family that contains a C‐terminal PRYSPRY domain that can bind with high affinity to the invariant chain, especially the Fc receptor of antibodies, endowing it with a distinctive role in antiviral responses and autoimmune diseases.^[^ ^37^, ^38^, ^39^ ^]^ Recently, TRIM21 has been found to participate in various biological processes, including tumorigenesis,^[^ ^40^, ^41^, ^42^ ^]^ pyroptosis.^[^ ^43^ ^]^ cell metabolism.^[^ ^44^ ^]^ and redox regulation.^[^ ^45^, ^46^ ^]^ However, the versatile biological function of TRIM21 remains elusive, especially in ferroptosis.

In this study, we found that FSP1 underwent K63 ubiquitination upon ferroptosis induction in GI tumors. Using immunoprecipitation‐mass spectrometry (IP‐MS), we identified TRIM21 as a bona fide partner of FSP1. TRIM21 mediated K63 ubiquitination of FSP1 on K322 and K366 residues, contributing to the membrane translocation and antiferroptotic function of FSP1. In vitro and in vivo experiments confirmed the antiferroptotic role of the TRIM21‐FSP1 axis. Furthermore, we explored the possibility of using ferroptosis inducers in pancreatic cancer treatment in the *Kras^LSL‐G12D/+^; Trp53 ^flox/flox^; Pdx1‐Cre* (KPC) mouse model. Our findings provide new insight into the mechanism by which cancer cells upregulate TRIM21 to promote the function of FSP1 to prevent ferroptosis and may shed light on ferroptosis‐related cancer therapy.

## 2 Results

### 2.1 Ferroptosis Inducers Promote the K63 Ubiquitination and Plasma Membrane Translocation of FSP1

**Figure 1:**
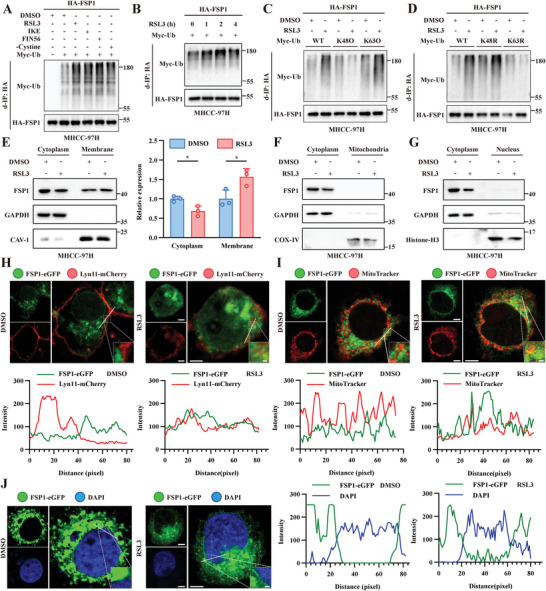
FSP1 undergoes K63 ubiquitination and plasma membrane translocation during ferroptosis. A) MHCC‐97H transfected with HA‐FSP1 and Myc‐ubiquitin were treated with different ferroptosis inducers including RSL3 (1 µM, 4 h), Imidazole Ketone Erastin (IKE, 1 µM, 4 h), FIN56 (1 µM, 4 h) or cystine deprivation culture medium (‐Cystine, 12 h). Cell lysates were subjected to denaturing‐immunoprecipitation (d‐IP) with anti‐HA antibody and analyzed by immunoblotting. (Related to Figure S1A, Supporting Information). B) MHCC‐97H cells transfected with HA‐FSP1 and Myc‐ubiquitin were treated with 1 µM RSL3 for 0–4 h. Cell lysates were analyzed by d‐IP using anti‐HA antibodies (Related to Figure S1C, Supporting Information). C‐D) MHCC‐97H cells transfected with HA‐FSP1 and Myc‐ubiquitin (wildtype, K48O, K63O, K48R or K63R) were treated with DMSO or 1 µM RSL3 for 4 h. Cells lysates were analyzed by d‐IP using anti‐HA antibodies (Related to Figure S1D,E, Supporting Information). E‐G) MHCC‐97H cells were treated with 1 µM RSL3 for 4 h prior to isolation of membrane (E), mitochondria (F) or nucleus (G) with cytosolic fractions. Immunoblotting was performed to evaluate the level of FSP1 in different fractions. *P* values were calculated by unpaired, two‐tailed Student's *t*‐test. *P < 0.05. H‐J) Confocal microscopy of MHCC‐97H cells transfected with FSP1‐eGFP and co‐transfected with Lyn11‐mCherry to label the plasma membrane (H), stained with 100 nM MitoTracker Red to label mitochondria (I) or stained with DAPI to label the nucleus (J). Line intensity plots show colocalization between FSP1‐eGFP and organelle markers. Fluorescent images are representative of at least *n* = 5 imaged cells. Scale bars, 5 µm.

To investigate the potential role of ubiquitination in FSP1 upon ferroptosis induction, human hepatocellular carcinoma cells MHCC‐97H and human pancreatic cancer cells PANC‐1 with stable FSP1 overexpression were transfected with Myc‐Ub and were treated with various ferroptosis inducers, including RSL3, imidazole ketone erastin (IKE), FIN56, and cystine‐deprived culture medium. There was an increase in FSP1 ubiquitination after ferroptosis induction (**Figure** **1**A and Figure S1A, B, Supporting Information), indicating that ubiquitination was probably involved in FSP1‐regulated ferroptosis. Considering that FSP1 was first identified to play an antiferroptotic role under GPx4 inhibition,^[^ ^10^, ^11^ ^]^ GPx4 inhibitor RSL3 was chosen as the ferroptosis inducer in the subsequent study. We further confirmed that the FSP1 ubiquitination level was gradually increased in a time‐dependent manner within 1–4 h RSL3 treatment (Figure 1B and Figure S1C, Supporting Information). The eight different linkages of ubiquitin chains lead to distinct molecular processes.^[^ ^20^ ^]^ To determine the exact type of FSP1 ubiquitination, ubiquitination assays were carried out using different Myc‐Ub mutants. There was a significant increase in ubiquitination in cells transfected with K63O and K48R ubiquitin upon RSL3 treatment, and this increase was abolished in cells transfected with K48O and K63R ubiquitin, suggesting that FSP1 underwent K63 ubiquitination upon ferroptosis induction (Figure 1C, D and Figure S1D,E, Supporting Information).

Previous studies indicated that the glycine‐2 to alanine (G2A) mutation in FSP1 abolished not only its ability to localize to the plasma membrane but also its ability to suppress ferroptosis, suggesting that the subcellular localization determines the function of FSP1.^[^ ^10^, ^11^ ^]^ To explore whether ferroptosis induction affects the subcellular localization of FSP1, different subcellular fractions of organelles were isolated. The subsequent immunoblot analysis confirmed the effect of RSL3 on the accumulation of FSP1 at the plasma membrane but not in the nucleus or mitochondria (Figure 1E‐G). To investigate the subcellular distribution of FSP1 more intuitively, confocal microscopy analysis was carried out in MHCC‐97H cells cotransfected with FSP1‐eGFP and Lyn11‐mCherry, which labeled the plasma membrane, or stained with MitoTracker to label the mitochondria or with DAPI to label the nucleus. Specifically, FSP1 showed increased colocalization with the plasma membrane (Figure 1H) but not with mitochondria (Figure 1I) or the nucleus (Figure 1J) after ferroptosis induction. Taken together, these results indicated that FSP1 underwent K63 ubiquitination and was translocated to the plasma membrane upon ferroptosis induction.

### 2.2 TRIM21 Binds to FSP1 through the PRY‐SPRY Domain

**Figure 2:**
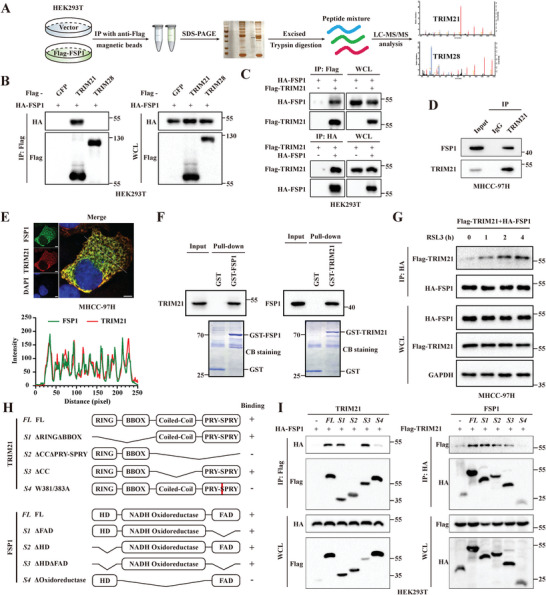
TRIM21 is a bona fide binding partner of FSP1. A) A schematic workflow of immunoprecipitation‐mass spectrometry (IP‐MS) experiments. B) HEK293T cells were transfected with HA‐FSP1 and Flag‐tagged GFP, TRIM21 or TRIM28 plasmids. Cell lysates were immunoprecipitated with anti‐Flag antibodies and analyzed using immunoblotting. C) HEK293T cells were transfected with indicated plasmids, followed by immunoprecipitation with anti‐HA or anti‐Flag antibodies and immunoblot with anti‐Flag or anti‐HA antibodies, respectively. D) MHCC‐97H cell lysates were collected, immunoprecipitated with anti‐TRIM21 antibodies, and immunoblotted with anti‐FSP1 antibodies. E) Confocal microscopy of TRIM21 (Red) and FSP1 (Green) in MHCC‐97H cells transfected with Flag‐TRIM21 and HA‐FSP1 plasmids and subjected to immunofluorescence assay. Line intensity plots shows colocalization between FSP1 and TRIM21. Images are representative of at least *n* = 5 imaged cells. Scale bars, 5 µm. F) Cell lysates from HEK293T cells were incubated with GST, GST‐FSP1 or GST‐TRIM21 conjugated to beads. Pull‐down samples and whole cell lysates were analyzed using immunoblotting and Coomassie blue staining. G) MHCC‐97H cells were co‐transfected with Flag‐TRIM21 and HA‐FSP1 and treated with 1 µM RSL3 for 0—4 h. Cell lysates were immunoprecipitated with anti‐HA antibodies and analyzed using immunoblotting. H) Schematic of the full‐length TRIM21, FSP1, and their truncated mutants. I) HEK293T cells were transfected with indicated full‐length or truncated mutants of TRIM21 or FSP1. Cell lysates were collected and subjected to immunoprecipitation with anti‐Flag or anti‐HA antibodies to explore the binding regions between TRIM21 and FSP1.

To identify the potential E3 ubiquitin ligase that mediates the K63 ubiquitination of FSP1, a systematic mass spectrometry analysis was conducted to identify FSP1‐interacting proteins by using HEK293T cells stably expressing Flag‐FSP1. The results showed that TRIM21 and TRIM28, members of the TRIM family of E3 ubiquitin ligases, were present in the FSP1 immunoprecipitate (**Figure** **2**A). Coimmunoprecipitation (Co‐IP) assays, which were conducted in HEK293T cells transfected with HA‐FSP1 and Flag‐TRIM21 or Flag‐TRIM28, confirmed the interaction between FSP1 and TRIM21 but not TRIM28 (Figure 2B). The binding of FSP1 to TRIM21 was further confirmed by both exogenous reciprocal co‐IP assays in HEK293T cells (Figure 2C) and endogenous co‐IP assays in MHCC‐97H cells (Figure 2D). Immunofluorescence staining followed by confocal analysis indicated the colocalization of FSP1 with TRIM21 in the cytoplasm of MHCC‐97H cells (Figure 2E). Furthermore, GST (glutathione S‐transferase)‐tagged FSP1 and GST‐tagged TRIM21 were expressed and purified from bacteria and incubated with protein lysates from MHCC‐97H cells for a GST pull‐down assay. The results confirmed that FSP1 could directly interact with TRIM21 (Figure 2F). Interestingly, there was a gradual increase in TRIM21 binding to FSP1 after 0–4 h of RSL3 treatment, indicating that TRIM21 might have a regulatory effect on FSP1 during ferroptosis induction (Figure 2G). A series of FSP1 and TRIM21 truncation mutants were constructed for co‐IP analysis, which indicated that the PRY‐SPRY domain of TRIM21, especially W381, and W383, was critical for TRIM21 interaction with the NADH oxidoreductase region of FSP1 (Figure 2H, I). Taken together, these results confirmed a bona fide interaction between FSP1 and TRIM21.

### 2.3 TRIM21 Mediates the K63 Ubiquitination of FSP1 at K322 and K366 Residues

**Figure 3:**
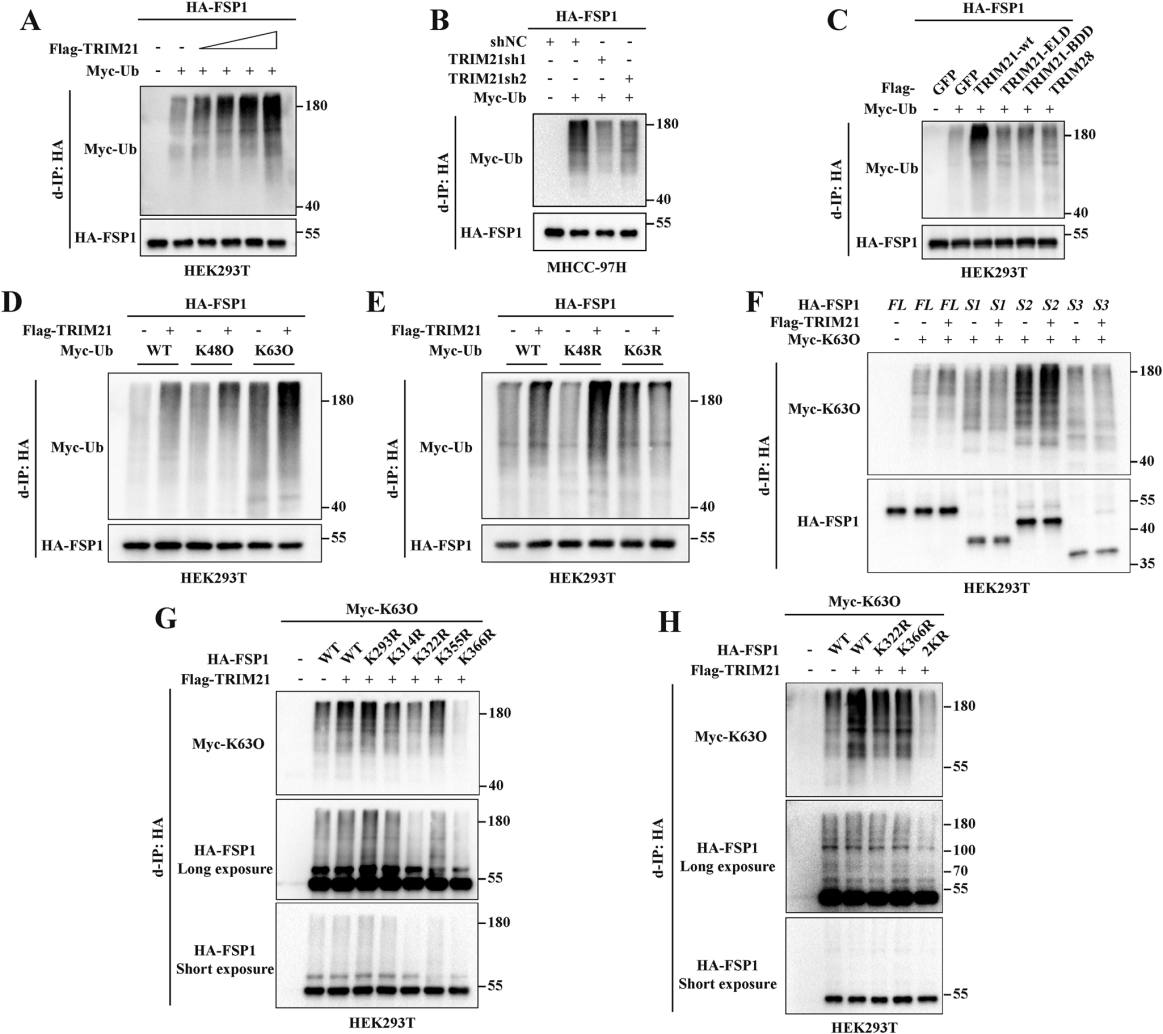
TRIM21 promotes the K63‐linked ubiquitination of FSP1 on K322 and K366 residues. A) HEK293T cells were transfected with HA‐FSP1, Myc‐ubiquitin, and increasing amounts of Flag‐TRIM21 as indicated. Cell lysates were analyzed by d‐IP using anti‐HA antibodies. (Related to Figure S2A). B) MHCC‐97H cells were transfected with HA‐FSP1, Myc‐ubiquitin, and shRNA targeting TRIM21. Cell lysates were analyzed by d‐IP using anti‐HA antibodies. (Related to Figure S2B, Supporting Information). C) HEK293T cells were transfected with HA‐FSP1, Myc‐ubiquitin, and Flag‐tagged wild type TRIM21 (TRIM21‐wt), or E3 ligase deficient TRIM21 (TRIM21‐ELD), or binding domain deficient TRIM21 (TRIM21‐BDD), or TRIM28. Cell lysates were analyzed by d‐IP using anti‐HA antibodies. (Related to Figure S2C, Supporting Information). D‐E) HEK293T cells were transfected with HA‐FSP1 and Myc‐ubiquitin (wild type, K48O, K63O, K48R, K63R), with or without co‐transfection of Flag‐TRIM21. Cell lysates were analyzed by d‐IP using anti‐HA antibodies. (Related to Figure S2D,E, Supporting Information). F) HEK293T cells were transfected with Myc‐Ubiquitin‐K63O, full‐length or truncated mutants of FSP1, with or without Flag‐TRIM21. Cell lysates were analyzed by d‐IP using anti‐HA antibodies. (Related to Figure S2G, Supporting Information). G) HEK293T cells were transfected with Myc‐Ubiquitin‐K63O and wild‐type or five lysine (K) to arginine (R) mutants of FSP1. Cell lysates were analyzed by d‐IP using anti‐HA antibodies. (Related to Figure S2H, Supporting Information). H) HEK293T cells were transfected with Myc‐Ubiquitin‐K63O and wild type, K322R, K366R or K322R/K366R (2KR) mutant of FSP1. Cell lysates were analyzed by d‐IP using anti‐HA antibodies. (Related to Figure S2I, Supporting Information).

To ascertain that TRIM21 promotes FSP1 ubiquitination, the ubiquitination assay was performed and indicated that ectopic expression of TRIM21 increased the ubiquitination of FSP1 in a dose‐dependent manner (**Figure** **3**A and Figure S2A, Supporting Information). In contrast, TRIM21 knockdown by shRNA inhibited the ubiquitination of FSP1 (Figure 3B and Figure S2B, Supporting Information). Moreover, both the E3 ligase‐deficient (ELD) and binding domain‐deficient (BDD) mutants of TRIM21 lost the ability to catalyze the ubiquitination of FSP1 (Figure 3C and Figure S2C, Supporting Information). Importantly, K63 ubiquitination of FSP1 was increased most significantly upon TRIM21 overexpression (Figure 3D, E and Figure S2D‐F, Supporting Information). To identify the critical FSP1 ubiquitination residues, Myc‐Ub and truncation mutants of FSP1 (see Figure 2H) were transfected with or without TRIM21. The ubiquitination assay showed that TRIM21 was unable to ubiquitinate FSP1 in FAD‐binding domain deletion mutants, indicating that the FAD binding domain was probably the region involved in FSP1 K63 ubiquitination (Figure 3F and Figure S2G, Supporting Information). All five lysine (K) residues in the FAD‐binding domain, i.e., K293, K314, K322, K355, and K366, were mutated to arginine (R) using site‐directed mutagenesis. Further performed ubiquitination assay showed that the K322R and K366R mutations drastically decreased the K63 ubiquitination of FSP1 mediated by TRIM21 (Figure 3G and Figure S2H, Supporting Information). Furthermore, compared with wild‐type FSP1, the combined mutation of K322 and K366 (FSP1‐2KR) almost completely abolished the K63 ubiquitination of FSP1 mediated by TRIM21 (Figure 3H and Figure S2I, Supporting Information). Collectively, these results suggested that TRIM21 was critical for K63 ubiquitination of K322 and K366 in FSP1 during ferroptosis induction.

### 2.4 TRIM21‐Mediated K63 Ubiquitination Promotes the Plasma Membrane Translocation of FSP1

**Figure 4:**
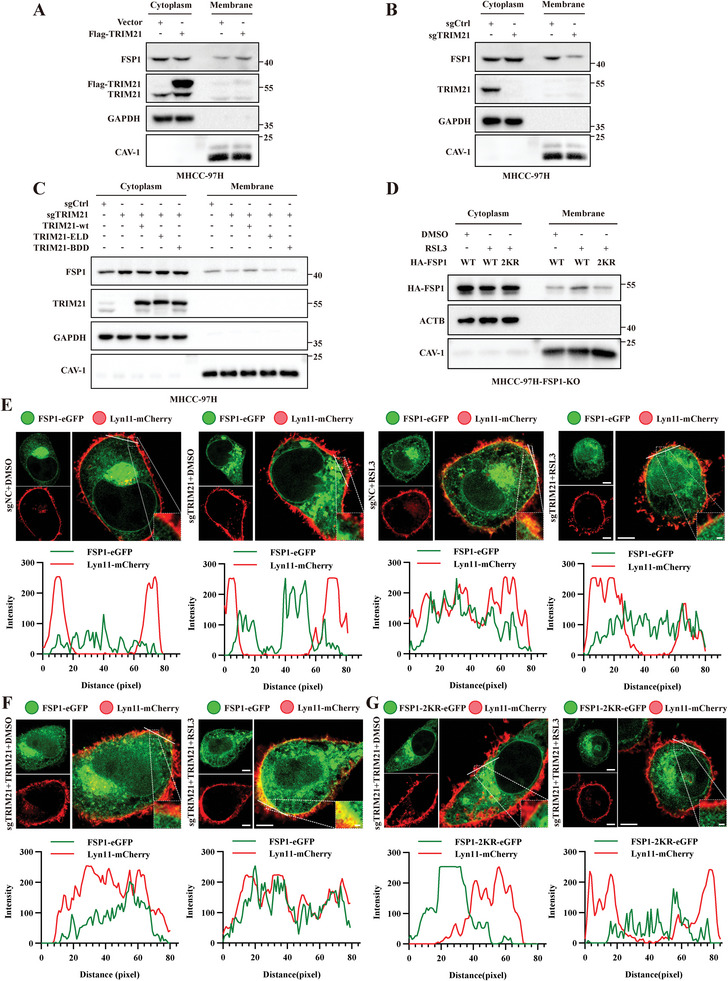
TRIM21 promotes the membrane translocation of FSP1 through K63 ubiquitination at K322 and K366 residues. A) MHCC‐97H cells transfected with vector or Flag‐TRIM21 were collected for isolation of membrane and cytosolic fractions. Immunoblotting was used to analyze the level of FSP1. B) TRIM21‐knockout and control MHCC‐97H cells were collected for isolation of membrane and cytosolic fractions. Immunoblotting was used to analyze the level of FSP1. C) TRIM21‐knockout MHCC‐97H cells were transfected with wildtype TRIM21 (TRIM21‐wt) or mutants of TRIM21 (TRIM21‐ELD, TRIM21‐BDD). Cell lysates were collected for the isolation of membrane and cytosolic fractions. Immunoblotting was used to analyze the level of FSP1. D) FSP1‐knockout MHCC‐97H cells were transfected with wildtype or K322R/K366R (2KR) mutant of HA‐FSP1, followed by DMSO or 0.1 µM RSL3 treatment for 4 h. Cell lysates were collected for isolation of membrane and cytosolic fractions. Immunoblotting was used to analyze the level of FSP1. E) Confocal microscopy of TRIM21‐knockout or control MHCC‐97H cells transfected with FSP1‐eGFP and Lyn11‐mCherry and treated with DMSO or 1 µM RSL3 for 4 h prior to fixing and photographing. F) Confocal microscopy of TRIM21‐knockout MHCC‐97H cells transfected with Flag‐TRIM21, Lyn11‐mCherry, and FSP1‐eGFP or FSP1‐2KR‐eGFP and treated with DMSO or 1 µM RSL3 for 4 h prior to fixing and photographing. Line intensity plots show colocalization between FSP1‐eGFP and the plasma membrane marker Lyn11‐mCherry. Fluorescent images are representative of at least *n* = 5 imaged cells. Scale bars, 5 µm.

To determine the effect of TRIM21‐mediated K63 ubiquitination on FSP1, we first ectopically expressed TRIM21 and monitored the expression of FSP1. The results indicated that TRIM21 did not affect the half‐life or stability of the FSP1 protein (Figure S3A‐F, Supporting Information). Our previous results indicated that ferroptosis induction promoted the K63 ubiquitination and plasma membrane translocation of FSP1. Thus, we speculated that TRIM21 promoted the translocation of FSP1 to the plasma membrane via K63 ubiquitination. To verify this hypothesis, membrane proteins and cytoplasmic proteins were separated and analyzed by immunoblotting. Our results indicated that TRIM21 overexpression promoted the translocation of FSP1 to the membrane, while knockout of TRIM21 using CRISPR/Cas9 gene editing had the opposite effect (**Figure** **4**A, B and Figure S4A, B, Supporting Information). To determine whether FSP1 translocation was associated with K63 ubiquitination, we re‐expressed wild‐type TRIM21, E3 ligase‐deficient TRIM21 mutants, and binding domain‐deficient TRIM21 mutants in TRIM21 knockout MHCC‐97H cells and found that wild‐type but not E3 ligase‐deficient or binding domain‐deficient TRIM21 rescued the membrane translocation of FSP1 (Figure 4C). Moreover, we transfected wild‐type FSP1 or the 2KR mutant of FSP1 into FSP1‐knockout MHCC‐97H cells and found that the 2KR mutation of FSP1 almost completely abolished its translocation to the membrane (Figure 4D). To demonstrate the translocation of FSP1 more intuitively, we transfected FSP1‐eGFP and Lyn11‐mCherry into MHCC‐97H cells and performed confocal microscopy analysis. Interestingly, ferroptosis inducer treatment promoted FSP1 translocation to the plasma membrane (Panel 3 vs. Panel 1, Figure 4E), which was abolished in TRIM21‐knockout cells (Panel 4 vs. Panel 2, Figure 4E). Overexpression of wild‐type TRIM21 restored the plasma membrane translocation of FSP1 (Figure 4F), but the FSP1‐2KR mutant remained in the cytoplasm despite RSL3 treatment and TRIM21 overexpression (Figure 4G). Taken together, these results indicated that TRIM21 facilitated the plasma membrane translocation of FSP1 by promoting K63 ubiquitination of K322 and K366 in FSP1.

### 2.5 The TRIM21‐FSP1 Axis Protects Tumor Cells Against Ferroptosis In Vitro

**Figure 5:**
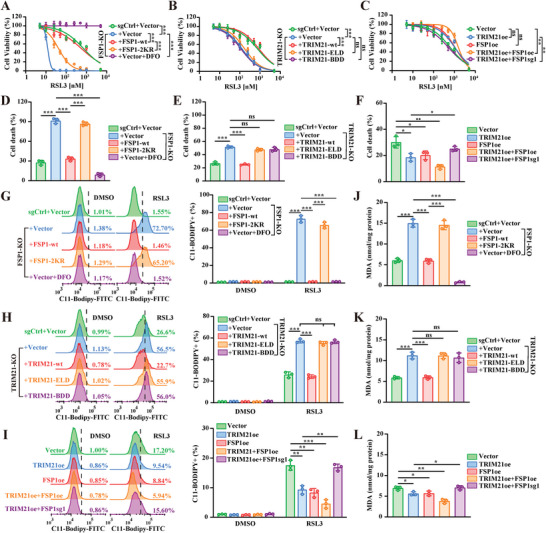
TRIM21 protects against ferroptosis through FSP1. A‐C) MHCC‐97H cells transfected with indicated plasmids were treated with 0–5 mM RSL3 for 24 h. Cell viability was evaluated by CCK‐8 assay. D‐F) MHCC‐97H cells transfected with indicated plasmids were treated with 0.1 µM RSL3 for 16 h. Cell viability was evaluated by LDH cytotoxicity assay. G‐I) MHCC‐97H cells transfected with indicated plasmids were treated with DMSO or 0.1 µM RSL3 for 1 h (G) or 1 µM RSL3 for 4 h (H and I) prior to C11‐BODIPY 581/591 staining. Flowcytometry were used to analyze the level of lipid peroxides as indicated by FITC fluorescence. Representative images of flowcytometry were shown in the left panel. C11‐BODIPY positive rate were counted in the right panel. J–L) MHCC‐97H cells transfected with indicated plasmids were treated with 1 µM RSL3 for 4 h. Lipid peroxides were measured using MDA assay. Data were presented as means ± SD; *n* = 3. *P* values were calculated by two‐way ANOVA (A‐C) or Student's *t*‐test (D‐L). *P < 0.05, **P < 0.01, ***P < 0.001, ns, not significant.

Given the important role of TRIM21 in promoting the membrane translocation of FSP1, we next assessed the biological role of TRIM21 in MHCC‐97H cells and in KPC‐1A cells established from ascites from the KPC mouse model. After treatment with the indicated concentrations of RSL3, Cell Counting Kit‐8 (CCK‐8) assays and LDH assays were used to evaluate cell viability and death, while C11‐BODIPY 581/591 staining and malondialdehyde (MDA) assays were used to evaluate lipid ROS levels. Consistent with previous studies, knockout of FSP1 robustly sensitized tumor cells to RSL3 and markedly increased the lipid ROS level; these effects were reversed by the ferroptosis inhibitor DFO. Interestingly, restoration of wild‐type FSP1, but not the FSP1‐2KR mutant, protected against RSL3‐induced cell death and lipid ROS accumulation (**Figure** **5**A, D, G, J and Figure S5A, Supporting Information). Moreover, knockout of TRIM21 sensitized tumor cells to RSL3, and this sensitization was reversed by overexpression of wild‐type TRIM21, but not the E3 ligase‐deficient or binding domain‐deficient mutant of TRIM21 (Figure 5B,E, H,K and Figure S5B,D, Supporting Information). Moreover, the overexpression of TRIM21 or FSP1 promoted cancer cell resistance to ferroptosis, and co‐overexpression had an additive effect. However, TRIM21 overexpression showed no protective effect in cells transfected with sgRNA targeting FSP1, indicating that FSP1 was a critical downstream effector of TRIM21 in ferroptosis regulation (Figure 5C,F,I,L and Figure S5C, Supporting Information). Meanwhile, we verified the influence of TRIM21 on the ferroptosis sensitivity of SW480 cells for colorectal tumor, SGC‐7901 cells for stomach tumor, and HuCC‐T1 cells for cholangiocarcinoma. Our results indicated that knockout of TRIM21 sensitized these tumor cells to ferroptosis induction (Figure S5E‐G, Supporting Information). Collectively, these results suggested that TRIM21 protected tumor cells against ferroptosis by promoting FSP1 ubiquitination at K322 and K366.

### 2.6 TRIM21 Ablation Sensitizes Tumor Cells to Ferroptosis In Vivo

**Figure 6:**
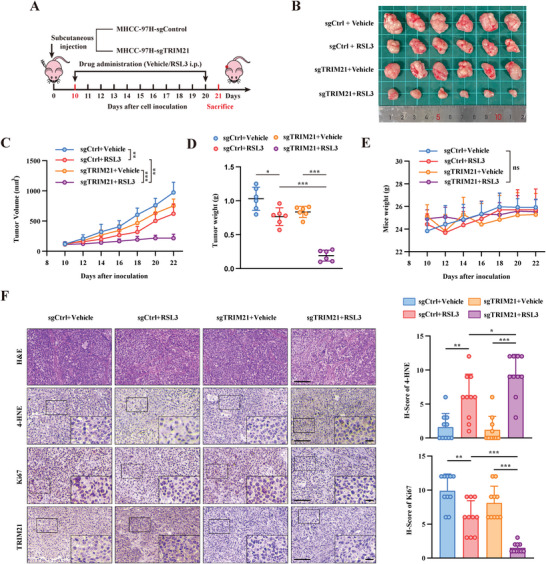
Knockout of TRIM21 sensitized cancer cells to ferroptosis. A) Schematic description of the experimental design of subcutaneous tumor model on nude mice. B) Images of resected subcutaneous tumors. C‐E) tumor volume (C), resected tumor weight (D), and mice weight (E) in the subcutaneous tumor model (*n* = 6). F) Representative images of H&E and immunohistochemistry staining of resected subcutaneous tumors from each group. H‐score of 4‐HNE and Ki67 were shown in the right panel (*n* = 10). Images are representative of at least *n* = 10 imaged sights. Scale bars, 100 µm. Data were presented as means ± SD. *P* values were calculated by two‐way ANOVA (C and E) or Student's *t*‐test (D and F). *P < 0.05, **P < 0.01, ***P < 0.001, ns, not significant.

To further evaluate the role of TRIM21 in ferroptosis, we established in vivo xenograft models by subcutaneously injecting control or TRIM21‐knockout MHCC‐97H cells into BALB/c nude mice. These tumor‐bearing mice were administered either vehicle or RSL3 (20 mg kg^−1^) every day beginning on day 10. All mice were sacrificed on day 21 after cell inoculation (**Figure** **6**A). Tumors from the RSL3 treatment group progressed at a much slower rate than the vehicle group, and this antitumor effect was further augmented in mice inoculated with TRIM21‐knockout MHCC‐97H cells (Figure 6B‐E). Moreover, RSL3 treatment resulted in lipid ROS accumulation and proliferation inhibition in xenograft tumors, as indicated by the elevated 4‐HNE and decreased Ki67 levels in xenograft tumor tissues. Consistent with the above results, these effects were further enhanced in mice inoculated with TRIM21‐knockout MHCC‐97H cells (Figure 6F). Thus, TRIM21 knockout contributed to the sensitization of tumor cells to ferroptosis in vivo.

### 2.7 TRIM21 is Highly Expressed in Multiple GI Tumors and is Further Upregulated Upon Ferroptosis Induction

**Figure 7:**
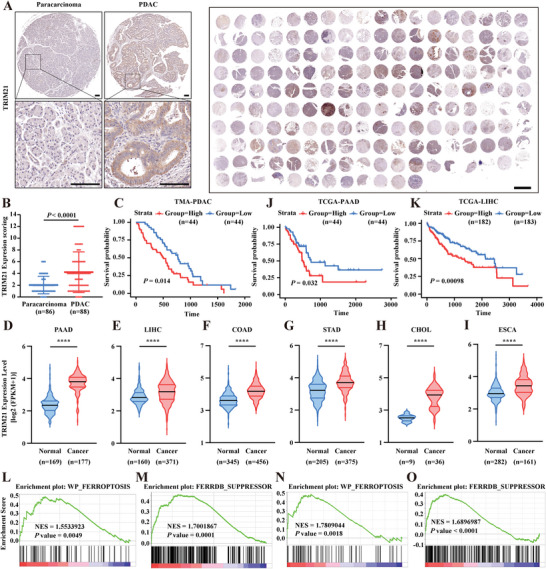
TRIM21 is highly expressed in GI tumors and is associated with poor prognosis. A) Immunohistochemistry staining of TRIM21 in human PDAC TMAs. Representative images are shown in the left panel. Scale bars represent 100 µm in left panel and 2 mm in the right panel. B) Scoring of TRIM21 expression based on PDAC TMAs in (A). Data were presented as means ± SD. *P* values were calculated by unpaired, two‐tailed Student's *t*‐test. C) Patients were divided into two groups based on the expression level of TRIM21. Kaplan‐Meier analysis was used to analyze the correlation between TRIM21 expression level and patients’ survival. D‐I) Expression of TRIM21 in GI tumors from TCGA database and corresponding normal tissues from GTEx database. (D) pancreatic cancer; (E) liver cancer; (F) colon cancer; (G) stomach cancer; (H) cholangiocarcinoma; (I) esophageal cancer. Data were presented as means ± SD. *P* values were calculated by unpaired, two‐tailed Student's *t*‐test. J‐K) Patients were divided into two groups based on expression level of TRIM21. Kaplan‐Meier analysis was used to analyze the correlation between TRIM21 expression level and patients’ survival in TCGA‐PAAD dataset (J) and TCGA‐LIHC dataset (K). L‐O) GSEA was conducted to explore the correlation between TRIM21 and ferroptosis related genes based on TCGA‐PAAD dataset (L and M) and GSE78229 dataset (N and O).

**Figure 8:**
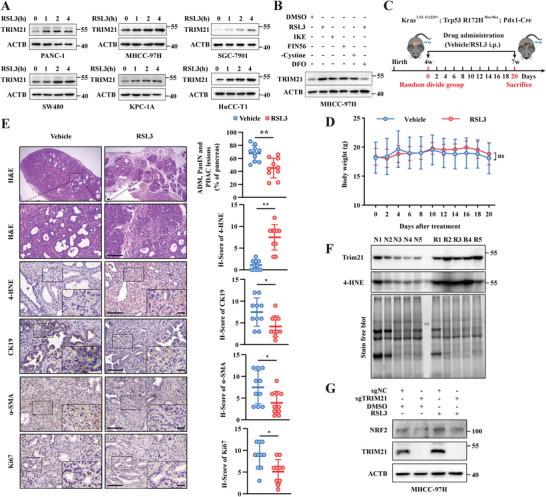
TRIM21 is stimulated upon ferroptosis induction. A) Expression level of TRIM21 in multiple GI tumor cell lines were determined by immunoblotting after 1 µM RSL3 treatment for 0–4 h. B) Expression level of TRIM21 in MHCC‐97H cells were determined by immunoblotting after RSL3 (1 µM, 4 h), IKE (1 µM, 4 h), FIN56 (1 µM, 4 h), cystine starvation culture medium (‐Cys, 12 h) and RSL3 (1 µM, 4 h) combined with DFO (100 µM, 4 h) treatment. C) Schematic description of the experimental design of ferroptosis induction in KPC mice model. D) Mice weight of KPC mice treated with vehicle (*n* = 5) or RSL3 (*n* = 5, 20 mg/kg i.p. q2d). E) Representative images of H&E and immunohistochemistry staining of pancreas resected from KPC mice treated with vehicle or RSL3. Percentage of acinar‐duct metaplasia (ADM), pancreatic intraepithelial neoplasia (PanIN), and PDAC lesions and H‐score of 4‐HNE, CK19, α‐SMA, and Ki67 were shown in the right panel. Scale bars, 100 µm. F) Levels of Trim21 and 4‐HNE in the pancreas from KPC mice treated with vehicle (N1‐N5) or RSL3 (R1‐R5) were determined by immunoblotting. G) Expression level of NRF2 were determined in TRIM21 knockout or control MHCC‐97H cells treated with DMSO or 1 µM RSL3 for 4 h. Data were presented as means ± SD. *P*‐values were calculated by two‐way ANOVA (D) and unpaired, two‐tailed Student's *t*‐test (E). *P < 0.05, **P < 0.01, ns, not significant.

Previous studies have noted that TRIM21 is highly expressed in various types of human cancers.^[^ ^47^, ^48^, ^49^, ^50^ ^]^ To determine the correlation of TRIM21 expression with pancreatic cancer, immunohistochemical (IHC) staining of PDAC tissue microarrays (TMAs) was performed, showing significant upregulation of TRIM21 in PDAC samples compared with paracarcinoma samples (**Figure** **7**A, B). In addition, Kaplan–Meier survival analysis revealed that the expression of TRIM21 was negatively correlated with the overall survival of PDAC patients (Figure 7C). By analyzing the RNA sequencing data of several major GI tumors in the TCGA database, including pancreatic, liver, colon, gastric, esophagus, and bile duct cancers, as well as the corresponding normal tissue data in the GTEx database, we found that TRIM21 was highly expressed in these 6 kinds of GI tumors (Figure 7D‐I). Consistent with the above findings, higher TRIM21 expression was also correlated with poor prognosis in pancreatic and liver cancer (Figure 7J, K). Next, GSEA was performed to explore whether TRIM21 expression was associated with ferroptosis. By analyzing RNA sequencing data from TCGA database and GSE78229 dataset, we found that the “WP_FERROPTOSIS” gene set and “FERRDB_SUPPRESSOR” gene set were enriched in the TRIM21‐high expression group in multiple GI tumors, indicating the potential regulatory role of TRIM21 in ferroptosis in GI tumors (Figure 7L‐O, Figure S6A‐E, Supporting Information). Friends' analysis further supported that TRIM21 probably functioned as a hub gene in ferroptosis‐related genes (Figure S6F). Furthermore, we evaluated the relationship between TRIM21 expression and ferroptosis in RSL3‐treated cancer cell lines and the KPC mouse model. By immunoblot analysis, we found that TRIM21 expression was gradually increased in a time‐dependent manner in RSL3‐treated PANC‐1, MHCC‐97H, SW480, KPC‐1A, SGC‐7901, and HuCC‐T1 cells during 0–4 h treatment (**Figure** **8**A). In addition, other ferroptosis inducers, including IKE, FIN56, and cystine‐deprived culture medium, also promoted TRIM21 expression, and the ferroptosis inhibitor DFO reversed this effect (Figure 8B). Next, 4‐week‐old KPC mice were randomly divided into two groups and intraperitoneally injected with either vehicle or 20 mg kg^−1^ RSL3 (Figure 8C). Consistent with previous studies,^[^ ^6^, ^51^ ^]^ the KPC mice injected with RSL3 did not lose body weight relative to those in the vehicle group, indicating that the injected dose was of biosafety (Figure 8D). However, RSL3 administration resulted in elevated levels of oxidative stress in PDAC tumor tissues of KPC mice, as indicated by the increase in 4‐HNE expression detected by immunohistochemistry and immunoblotting (Figure 8E,F). Importantly, RSL3 administration also significantly slowed the progression of PDAC, as reflected by the decrease in the CK19‐positive area, α‐SMA‐positive area, and a number of Ki67‐positive cells. Consistent with the results of in vitro experiments, the expression of TRIM21 was significantly increased in RSL3‐treated KPC mice (Figure 8F). Previous studies showed that TRIM21 may augment oxidative stress through the p62‐KEAP1‐NRF2 pathway.^[^ ^45^, ^46^, ^50^ ^]^ However, knockout of TRIM21 did not affect NRF2 expression in RSL3‐treated cancer cells (Figure 8G). Together, these results indicated that high expression of TRIM21 in multiple GI tumors was associated with poor patient prognosis and that the TRIM21 expression level was elevated further in response to ferroptosis inducer treatment.

## 3 Discussion

Ferroptosis is regarded as a vulnerability in cancer due to the distinctive metabolism and high level of ROS in cancer cells.^[^ ^5^ ^]^ On the other hand, cancer cells exploit their ferroptosis defense systems, including upregulation of ferroptosis suppressor proteins, among which GPx4 is the most important and canonical,^[^ ^3^, ^52^ ^]^ to overcome this susceptibility. GPx4 uses GSH to neutralize lipid peroxides and prevent ferroptosis. Inhibition of GPx4 commonly leads to cell death due to excessive lipid ROS accumulation.^[^ ^53^, ^54^ ^]^ However, some cancer cells are resistant to ferroptosis even without GPx4 inhibition, indicating the existence of alternative ferroptosis defence mechanisms.^[^ ^6^ ^]^ In this situation, FSP1 was discovered as a vital ferroptosis suppressor acting in parallel to GPx4, which facilitates the generation of CoQH_2_ or VKH_2_ by the use of NAD(P)H on the plasma membrane.^[^ ^10^, ^11^, ^12^ ^]^ However, the posttranslational modifications of FSP1, especially ubiquitination, are still poorly understood. In the present study, we discovered a novel posttranslational modification of FSP1, K63 ubiquitination, that promoted FSP1 translocation to the plasma membrane upon ferroptosis induction. We identified TRIM21 as the E3 ligase that bound to FSP1 and catalyzed its K63 ubiquitination at K322 and K366, which had no influence on the half‐life and stability of the FSP1 protein but promoted its plasma membrane translocation and enhanced its ferroptosis suppression ability. In vitro and in vivo functional experiments confirmed the role of the TRIM21‐FSP1 axis in ferroptosis resistance. Additionally, we determined that TRIM21 was highly expressed in multiple GI tumors and was further upregulated upon ferroptosis induction. Our study reveals that cancer cells respond to and inhibit ferroptosis by upregulating TRIM21 to promote FSP1 membrane translocation and FSP1 function through K63 ubiquitination.

Previous studies have emphasized the importance of plasma membrane localization to the function of FSP1. The G2A mutation of FSP1, which abolishes G2 myristoylation, results in the inability of FSP1 to localize to the plasma membrane and suppress ferroptosis. Retargeting of FSP1‐G2A to the plasma membrane, but not to the endoplasmic reticulum, mitochondria or lipid droplets, restored its function of suppressing ferroptosis.^[^ ^10^, ^11^ ^]^ However, the subcellular distribution of FSP1 upon ferroptosis induction remains unknown. Our results suggested that FSP1 underwent K63 ubiquitination and translocated to the plasma membrane but not to mitochondria or the nucleus after RSL3 treatment. Moreover, we found that K322 and K366 are important residues for FSP1 K63 ubiquitination, whereas mutations at these residues abolish the plasma membrane localization and antiferroptotic effect of FSP1. Our study established a connection between ferroptosis induction and PTMs and the subsequent distribution of ferroptosis‐related proteins. Studies have indicated that unlike proteolytic K48 ubiquitination, K63 ubiquitination may promote or prevent the plasma membrane translocation of the target protein.^[^ ^30^, ^31^, ^55^ ^]^ Here, we found that the K63 ubiquitination level of FSP1 together with its plasma membrane localization was increased upon ferroptosis induction. Most importantly, FSP1‐2KR mutant lost the ability of plasma membrane translocation and lipid peroxides elimination in response to ferroptosis induction. Thus, we inferred that PDAC cells exploited the FSP1‐CoQH2/VKH2 system by transferring FSP1 to the plasma membrane with the assistance of K63 ubiquitination, facilitating the elimination of lipid peroxides when the GPx4‐GSH system was inhibited. Whether the C‐terminal K63 ubiquitination and N‐terminal myristoylation of FSP1 are relevant for its plasma membrane transport remains unknown. Further studies are needed to investigate whether this K63 ubiquitination is involved in the subcellular distribution and function of FSP1 by affecting FSP1 G2 myristoylation.

Emerging evidence has shown that TRIM21 plays dual roles in cancer. TRIM21 inhibited the proliferation, migration, and invasion of breast cancer cells by mediating the ubiquitination and proteasomal degradation of SET7/9.^[^ ^41^ ^]^ TRIM21 was also found to promote the ubiquitination of mutant p53 to suppress tumorigenesis.^[^ ^42^ ^]^ Additionally, TRIM21‐mediated G6PD degradation inhibited PI3K/AKT‐induced pentose phosphate pathway activation, thereby suppressing the proliferation of cancer cells.^[^ ^44^ ^]^ On the other hand, TRIM21 promoted colorectal cancer cell proliferation by promoting K63 ubiquitination of CLASPIN to inhibit CHK1 activation.^[^ ^48^ ^]^ TRIM21 was also found to promote cisplatin resistance in colon and pancreatic cancer cells through the downregulation of Par‐4 expression.^[^ ^56^ ^]^ As mentioned before, TRIM21 promotes p62 ubiquitination and abolishes the sequestration of Keap1 by p62, which results in the degradation of NRF2 and the elevation of oxidative stress levels.^[^ ^45^ ^]^ Knockout of TRIM21 leads to protection against arsenic‐induced liver injury and heart damage induced by transverse aortic constriction.^[^ ^45^ ^]^ More importantly, TRIM21 knockout was also found to protect the myocardium from doxorubicin‐induced ferroptosis in a mouse model.^[^ ^46^ ^]^ However, our results suggested that TRIM21 protected cancer cells from RSL3‐induced ferroptosis by promoting the K63 ubiquitination and plasma membrane translocation of FSP1 but not through p62‐NRF2 axis‐induced oxidative stress. Since different cells usually possess different metabolic features, they may exhibit distinct stress response characteristics upon stimulation. Therefore, these seemingly contradictory results may arise from the different models and treatments used in experiments. In addition, TRIM21 was highly expressed in multiple GI tumors and was associated with poor prognosis in PDAC and hepatocellular carcinoma. Our study indicated that TRIM21 was further upregulated upon ferroptosis induction in cancer cells and PDAC mouse models. Cancer cells are intrinsically more susceptible to ferroptosis due to their iron and lipid hypermetabolism and higher ROS levels compared to normal cells;^[^ ^4^, ^5^ ^]^ thus, we hypothesized that cancer cells could upregulate TRIM21 under conditions of high oxidative stress. Although high concentrations of ferroptosis inducers can increase drug concentrations at tumor sites and inhibit tumor growth, they may also have adverse effects on normal tissues and cells.^[^ ^57^, ^58^ ^]^ Some specific E3 ligases, represented by TRIM21, have high expression patterns and ferroptosis responsiveness in cancer. Combining E3 ligase inhibitors with existing ferroptosis inducers at appropriate concentrations may possess synergistic tumor‐suppressive effects. Further investigations are warranted to discover TRIM21‐targeting inhibitor.

In conclusion, our study demonstrates the importance of the TRIM21‐FSP1 axis in ferroptosis resistance in cancer cells. Targeting this process may induce ferroptosis in GI tumors and provide a new strategy for cancer treatment.

## 4 Experimental Section

The reagents are described in Table S1, Supporting Information.

### Patient samples

Human PDAC samples and paracarcinoma samples were collected from patients who underwent pancreatic surgery in the Department of Biliary‐Pancreatic Surgery at Tongji Hospital of Tongji Medical College of Huazhong University of Science and Technology (Wuhan, China). Informed consent forms were signed in advance. Follow‐up was conducted in time to record the survival time of patients. All studies associated with clinical samples and data were carried out in accordance with the principles of the Declaration of Helsinki and were approved by the Ethics Committee of Tongji Hospital, Tongji Medical College, Huazhong University of Science and Technology.

### Construction of the KPC mouse model and generation of the KPC‐1A cell line

Kras^LSL‐G12D/+^ mice (008179#), Trp53 ^flox/flox^ mice (008462#), and Pdx1‐Cre mice (014647#) were purchased from The Jackson Laboratory (ME, USA). Kras^LSL‐G12D/+^ and Pdx1‐Cre mice were crossed with Trp53^flox/flox^ mice. After two generations, Kras^LSL‐G12D/+^; Trp53^flox/flox^ mice and Pdx1‐Cre; Trp53^flox/flox^ mice were crossed to generate Kras^LSL‐G12D/+^; Trp53^flox/flox^; Pdx1‐Cre (KPC) mice. To generate the KPC‐1A cell line, the ascites from a 7‐week‐old male KPC mouse was collected by a sterilized injector and centrifuged at 1000 rpm for 5 min. After washing with PBS twice, the remaining cells were cultured with Dulbecco's modified Eagle's medium (DMEM) with 10% fetal bovine serum (FBS), 100 U mL^−1^ penicillin, and 100 µg mL^−1^ streptomycin. After subculturing for 3 generations, a stable adherent cell line was obtained and named KPC‐1A.

### Cell Culture

The HEK293T, MHCC‐97H, PANC‐1, SW480, A549, Hela, and KPC‐1A cells were cultured in DMEM while HuCC‐T1 and SGC‐7901 cells were cultured in RPMI‐1640 with 10% FBS and penicillin‒streptomycin at 37 °C in a humidified atmosphere of 5% CO_2_. HEK293T, PANC‐1, SW480, A549, and Hela cell lines were from ATCC, and MHCC‐97H, HuCC‐T1, and SGC‐7901 cell lines were purchased from Meisen (Zhejiang, China). All cell lines were confirmed to be mycoplasma free before experiments were performed.

### Plasmid Construction, Transfection, and Lentivirus Production

Flag‐FSP1, Flag‐TRIM21, Flag‐TRIM28, and HA‐FSP1 were generated by cloning human FSP1 cDNA and TRIM21 or TRIM28 cDNA into the pHAGE‐Flag or pHAGE‐HA vector, respectively. Point mutated or truncated fragments of TRIM21 or FSP1 were generated by PCR using the plasmids containing full‐length Flag‐TRIM21 or HA‐FSP1, respectively, as templates. GST‐FSP1 and GST‐TRIM21 were generated by inserting human FSP1 and TRIM21 cDNA, respectively, into the pGEX‐4T1 vector. FSP1‐eGFP was generated by insertion of a linker sequence (GSAGSAAGSGEF) and eGFP downstream of the coding sequence of FSP1. Lyn11 is the first 11 amino acids of Lyn kinase (MGCIKSKGKDS) that contains myr‐palm tag (MGCIKSK), which encodes a peptide for myristoylation and palmitoylation that is sufficient to target a protein to the plasma membrane.^[^ ^59^ ^]^ Lyn11‐mCherry was generated by insertion of Lyn11 and a linker sequence (RSANSGAGAGAGAILSR) upstream of the coding sequence of mCherry. ShRNA of TRIM21 (Clone ID: TRCN0000003983, TRCN0000234748) was synthesized by Sangon Biotech (Shanghai, China) and subcloned into the pLKO.1 vector. The sequences of all primers used were summarized in Table S2, Supporting Information. All plasmids were verified by Sanger sequencing before usage. Transfection was performed using polyethyleneimine (PEI, Sigma) according to the manufacturer's instructions. For lentivirus production, HEK293T cells were cotransfected with the lentiviral plasmid and the packaging/envelope plasmids (psPAX2 and pMD2.G), and the medium was replaced with fresh medium 6 h after transfection. Viral supernatants were collected 72 h after transfection, centrifuged at 1000 rpm for 5 min, and filtered through a 0.45 µm filter (Millipore, MA, USA). Target cells were infected with viral supernatant in the presence of polybrene (Yeasen, Shanghai, China) for 8 h and selected for at least one week with 1 µg mL^−1^ puromycin 48 h after infection.

### CRISPR‒Cas9‐Mediated Gene Editing

To generate FSP1 and TRIM21 knockout MHCC‐97H cell lines, a single guide RNA (sgRNA) targeting human FSP1 or human TRIM21 was ligated into the PX459 vector. To generate the KPC‐1A cell line, a sgRNA targeting mouse Fsp1 or mouse Trim21 was designed. Cells were transfected with the corresponding sgRNA. After 48 h, 1 µg mL^−1^ puromycin was added to the culture medium for 3 days. The selected cells were then seeded in 96‐well plates for isolating single colonies. Clones were identified by Sanger sequencing and confirmed by immunoblot analysis.

### Co‐Immunoprecipitation and Denaturing IP

For co‐IP, cells were collected and lysed in 1 mL IP lysis buffer (20 mM Tris‐HCl at pH 7.4, 1 mM EDTA, 150 mM NaCl, and 1% NP‐40, supplemented with cOmplete protease inhibitor cocktail (Roche, Basel, Switzerland)) and sonicated on ice. After centrifugation at 12 000 rpm for 15 min, 50 µL of the supernatant was collected and boiled for 10 min with loading buffer as the whole‐cell lysate. Next, the indicated antibody and Protein A/G magnetic beads were added to the remaining supernatant and incubated at 4 °C overnight. The beads were washed three times with IP lysis buffer and boiled with 2 × loading buffer for 10 min. Beads were removed by centrifugation, and the precipitated samples were subjected to immunoblot analysis as previously reported.^[^ ^60^ ^]^

Denaturing IP was used for detecting ubiquitination. In brief, cells were treated with 10 µM MG132 for 6 h, collected by centrifugation, lysed in 90 µL of IP lysis buffer supplemented with 10 µL of SDS solution (10% w/v), and boiled at 95 °C for 5 min. Cell lysates were then diluted tenfold by adding 900 µL of IP lysis buffer and sonicated on ice. The following steps were the same as those described above.

### GST Pull‐Down Assay

For the GST pull‐down assay, proteins purified from HEK293T cells were incubated with GST magnetic beads bound to bacterially expressed GST or GST fusion proteins in GST lysis buffer (50 mM Tris‐HCl at pH 7.4, 0.5% NP‐40, 1 mM EDTA, 150 mM NaCl, protease inhibitor cocktail) at 4 °C overnight. Beads were washed three times and boiled with 2 × loading buffer for 10 min. The precipitated samples and input were analyzed by immunoblotting.

### Isolation of Subcellular Fractions and Proteins

Membrane proteins, mitochondrial proteins, nuclear proteins, and cytoplasmic proteins were isolated using subcellular fractionation protein extraction kits (P0033, C3601, P0028, Beyotime, Shanghai, China) according to the manufacturer's instructions. Proteins were analyzed by SDS‒PAGE and immunoblotting.

### Immunofluorescence Staining and Fluorescence Microscopy

For immunofluorescence staining, cells grown on coverslips were transfected with HA‐FSP1 and Flag‐TRIM21. After 48 h, the cells were washed twice with PBS and fixed for 15 min in 4% (w/v) paraformaldehyde. After permeabilization with 0.1% Triton X‐100 for 10 min, the cells were blocked with 5% BSA at 37 °C for 15 min, incubated with primary antibodies at 4 °C overnight, and incubated with secondary antibodies for 1 h at room temperature. After washing with PBS three times, the coverslips were mounted on glass slides using antifade mounting medium (G1401, Servicebio, Wuhan, China). For fluorescence microscopy of Lyn11‐mCherry and FSP1‐eGFP, cells were transfected with the indicated plasmid. After 48 h, the cells were fixed with 4% (w/v) paraformaldehyde for 15 min and washed with PBS 3 times prior to mounting. Immunofluorescence images were acquired on a confocal microscope (LSM780, Carl Zeiss, Germany). The fluorescence intensity of each channel was quantified by ImageJ software and was converged into curves using GraphPad Prism 9. Colocalization was determined by the degree of overlap between curves.

### Lipid Peroxidation Analysis by C11‐BODIPY

For lipid ROS detection, cells were treated as indicated and washed with PBS before incubation with 2 µM C11‐BODIPY 581/591 (D3861, Invitrogen, CA, USA) for 15 min in a humidified incubator (37 °C, 5% CO_2_). Cells were washed with PBS, trypsinized, and collected for analysis by a flow cytometer (CytoFLEX, Beckman, IN, USA). Lipid ROS‐positive cells were identified as those with a fluorescence level exceeding 99% of that in the control group.

### Malondialdehyde (MDA) Measurement

MDA levels were measured by a lipid peroxidation MDA assay kit (S0131, Beyotime) as previously reported.^[^ ^61^ ^]^ In brief, cells or tissues were collected and lysed with RIPA lysis buffer. After sonication and centrifugation, the suspension was collected, and the concentration of protein was determined by a BCA protein assay kit (P0011, Beyotime). Thiobarbituric acid (TBA) was added to the remaining supernatant and heated at 100 °C for 15 min to form MDA‐TBA adducts. After centrifugation at 12 000 rpm for 10 min, the supernatant was added to a 96‐well plate, and the absorbance at 532 nm was measured using a microplate reader (BioTek Instruments, VT, USA). MDA levels were normalized to milligrams of protein and compared between groups.

### Transmission Electron Microscopy (TEM)

TEM was conducted for the direct observation of cell morphology during ferroptosis. After treatment, MHCC‐97H cells were washed with PBS twice and collected by centrifugation. Cells were resuspended in Electron Microscope Fixative (G1102, Servicebio) and fixed overnight. After washing with PBS, cells were fixed by 1% OsO4 for 2 h, followed by dehydration using ethanol and acetone series. After being embedded in epoxy resin, the samples were cut into sections of 50 nm and stained with lead citrate. Pictures were examined using a transmission electron microscope (HT7800, Hitachi).

### Cell viability and death assays

For the cell viability and death assays, 4000 cells were seeded in 96‐well plates and allowed to grow overnight before treatment with the indicated concentration of RSL3 for 24 h. Viability was assessed using CCK‐8 reagent (CK04, Dojindo, Kumamoto, Japan). After treatment, the cell culture medium was replaced with 100 µL fresh medium containing 10 µL CCK‐8 solution and incubated for 1–4 h in a humidified incubator (37 °C, 5% CO_2_). The absorbance at 450 nm was measured with a microplate reader. Cell death was quantified by a cytotoxicity LDH assay kit (CK12, Dojindo). After treatment, 100 µL working solution was added to each well and incubated at 37°C for 30 min. Fifty microlitres of stop solution was added to each well, and the absorbance at 490 nm was measured.

### In Vivo Experiment

Animal studies were approved by the Laboratory Animal Welfare and Ethics Committee of Tongji Hospital, Tongji Medical College, Huazhong University of Science and Technology (Approval number: TJH‐202207012). For the subcutaneous transplantation tumor model, 6‐week‐old female BALB/c nude mice were obtained from GemPharmatech (Jiangsu, China). TRIM21 knockout or control MHCC‐97H cells (2 × 10^6^ per mouse) were injected into the right axillae of nude mice (*n* = 12 per group), and tumors were allowed to grow for 10 days. RSL3 was dissolved in the vehicle (5% DMSO + 40% PEG 300 + 5% Tween 80 + 50% double distilled H_2_O). Mice were then randomly separated into four groups: 1) sgControl + vehicle (*n* = 6); 2) sgControl + RSL3 (*n* = 6); (3) sgTRIM21 + vehicle (*n* = 6); and (4) sgTRIM21+ RSL3 (*n* = 6). The mice in these groups were treated with 20 mg/kg RSL3 or equal amounts of vehicle i.p. every day for 10 days. The weights of the mice and volumes of the xenograft tumors (calculated as 1/2 × length × width^2^) were determined every two days. At the end of the treatment, all mice were sacrificed, and the xenograft tumors were weighed, the tumor volumes were calculated, and the tumors were collected and fixed with 4% paraformaldehyde.

For the KPC mouse model, 10 male KPC mice were randomly divided into two groups at 4 weeks of age and injected with 20 mg kg^−1^ RSL3 or an equal amount of vehicle i.p. every two days for 3 weeks. The body weights of the mice were measured every two days. Mice were sacrificed at day 20 after injection, and the pancreases were either fixed with 4% paraformaldehyde for histological analysis or cut into small pieces with a mass of ≈30 mg, immersed in liquid nitrogen for snap freezing, and stored at −80 °C for RNA and protein extraction.

### Histology and Immunohistochemistry

Tissues were embedded in paraffin and cut into 5 µm sections. The sections were dewaxed, rehydrated, and stained with haematoxylin and eosin (H&E) or boiled in pH 9.0 EDTA antigen retrieval solution (MVS‐0099, MXB Biotechnologies, Fuzhou, China) for 30 min. After 3% H_2_O_2_ treatment for 30 min to quench endogenous peroxidase activity, the sections were washed with PBS, blocked with 5% BSA and incubated with the indicated primary antibodies at 4 °C overnight. After washing with PBS 3 times, the sections were incubated with a horseradish peroxidase (HRP)‐linked secondary antibody, and color development was performed using a DAB kit (DAB‐1031, MXB Biotechnologies).

For TMA analysis, TRIM21 expression in a human PDAC TMA was evaluated and quantitatively scored by two experienced pathologists according to the percentage of positive cells and the staining intensity. The percentage of positive cells was scored from 0 to 4: 0, 0–5%; 1, 6%‐25%; 2, 26%‐50%; 3, 51%‐75%; and 4, 76%‐100%. The staining intensity was scored from 0 to 3: 0, negative; 1, weak; 2, moderate; and 3, strong. The final scores were calculated as score (percentage of positive cells) × score (staining intensity) and averaged from 3 random visual fields.

### Bioinformatics

For TRIM21 expression and survival analysis, gene expression profiles (in FPKM) and clinical information of patients with various cancer types, including pancreatic cancer (PAAD), liver cancer (LIHC), colon cancer (COAD), stomach cancer (STAD) and bile duct cancer (COAD), in The Cancer Genome Atlas (TCGA) database were downloaded from the UCSC Xena website (https://xenabrowser.net/). Expression profiles of normal tissues in the Genotype‐Tissue Expression (GTEx) database were also downloaded from UCSC Xena, and GraphPad Prism 9 was used for expression analysis. Survival analysis was conducted using the “survival” and “survminer” packages in R 4.0.3.

For gene set enrichment analysis (GSEA), the GSE78229 dataset was downloaded from the Gene Expression Omnibus (GEO) website (https://www.ncbi.nlm.nih.gov/geo/). Genes related to ferroptosis suppression were downloaded from the FerrDB database (http://www.zhounan.org/ferrdb/current/)^[^ ^62^ ^]^ and named the “FERRDB_SUPPRESSOR” gene set. The “WP_FERROPTOSIS” gene set from the GSEA website^[^ ^63^ ^]^ (http://www.gsea‐msigdb.org/gsea/msigdb/human/genesets.jsp) was also chosen to explore the associations between TRIM21 and ferroptosis‐related genes. The lists of genes in the two gene sets are shown in Table S3, Supporting Information. GSEA was conducted using GSEA 4.3.2 software.^[^ ^64^, ^65^ ^]^ Friends analysis was conducted using GOSemSim package in R^[^ ^66^ ^]^ to explore whether TRIM21 functions as an important hub gene in WP_FREEOPTOSIS gene sets.

### Statistical Analysis

Statistical analysis was performed using GraphPad Prism 9. Each experiment was performed at least three times unless otherwise indicated. Data are presented as the means ± SDs. Two‐way ANOVA or two‐tailed, unpaired Student's *t*‐test was used for group comparisons unless otherwise indicated.

## Conflict of Interest

The authors declare no conflict of interest.

## Author Contributions

J.G. and Y.L. contributed equally to this work. J.G., Y.L. and X.L. designed the experiments; J.G. and Y.L. performed most of the experiments with the assistance from W.W., R.H., Q.X., L.C., Y.G., Y.S., Y.B., Q.Z. and Y.L.; J.G. and Y.L. wrote and edit the manuscript; R.H., M.W. and R.Q. provided funding support for the project; F.Z., M.W., X.L. and R.Q. performed data analysis and supervised the project. All authors read and approved the final paper.

## Supporting information

Supporting Information

## Data Availability

The data that support the findings of this study are available from the corresponding author upon reasonable request.
