# Supplementary material for: TRIM21‐Promoted FSP1 Plasma Membrane Translocation Confers Ferroptosis Resistance in Human Cancers
Source: Adv Sci (Weinh). 2023 Aug 16;10(29):2302318. doi: 10.1002/advs.202302318 (PMC10582465; doi:10.1002/advs.202302318)
Supplement: Supplementary file 1 — Supporting Information [file ADVS-10-2302318-s001.pdf]

ADVANCED SCIENCE
Open Access

Supporting Information

for *Adv. Sci.*, DOI 10.1002/advs.202302318

TRIM21-Promoted FSP1 Plasma Membrane Translocation Confers Ferroptosis Resistance in Human Cancers

Jun Gong, Yuhui Liu, Wenjia Wang, Ruizhi He, Qilong Xia, Lin Chen, Chunle Zhao, Yang Gao, Yongkang Shi, Yu Bai, Yangwei Liao, Qi Zhang, Feng Zhu, Min Wang*, Xu Li* and Renyi Qin**

Supporting Information

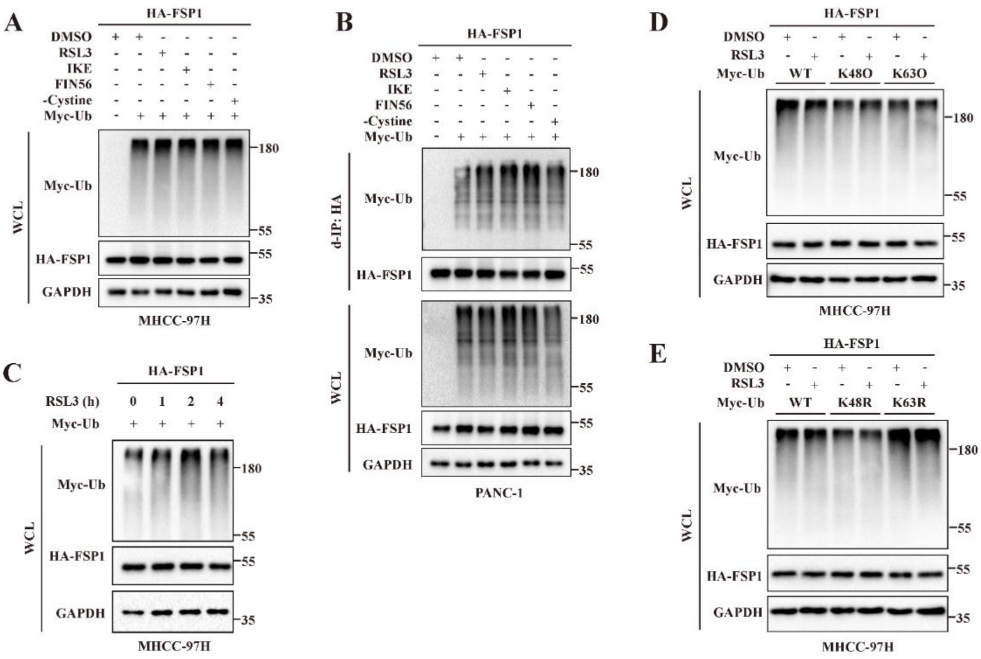

Figure S1. FSP1 undergoes K63-linked ubiquitination and plasma membrane translocation during ferroptosis. (See also: **Figure 1**). A-B) MHCC-97H (A) and PANC-1 (B) cells transfected with HA-FSP1 and Myc-ubiquitin were treated with different ferroptosis inducers including RSL3 (1 μM, 4 h), Imidazole Ketone Erastin (IKE, 1 μM, 4 h), FIN56 (1 μM, 4 h) or cystine deprivation culture medium (-Cystine, 12 h). Cell lysates were analyzed by d-IP using anti-HA antibodies. WCL: whole cell lysates. C) MHCC-97H cells transfected with HA-FSP1 and Myc-ubiquitin were treated with 1 μM RSL3 for 0-4 h. Whole cell lysates were analyzed by immunoblotting. D-E) MHCC-97H cells transfected with HA-FSP1 and Myc-ubiquitin (wildtype, K48O, K63O, K48R or K63R) were treated with DMSO or 1 μM RSL3 for 4 h. Whole cell lysates were analyzed by immunoblotting.

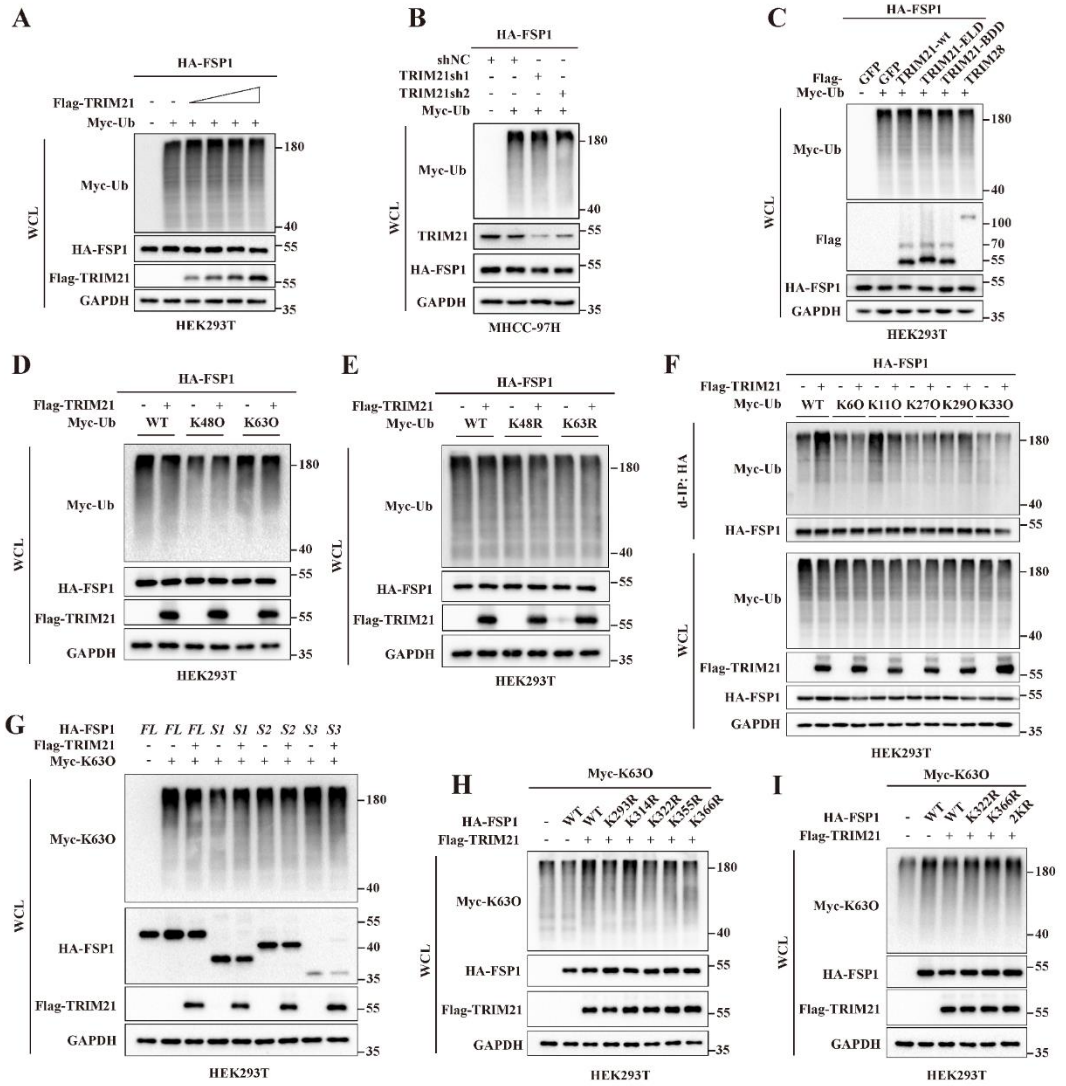

Figure S2. TRIM21 promotes the K63-linked ubiquitination of FSP1 on K322 and K366 residues. (See also: **Figure 3**). A) HEK293T cells were transfected with HA-FSP1, Myc-ubiquitin and increasing amounts of Flag-TRIM21 as indicated. Whole cell lysates were analyzed by immunoblotting. B) MHCC-97H cells were transfected with HA-FSP1, Myc-ubiquitin and shRNA targeting TRIM21. Whole cell lysates were analyzed by immunoblotting. C) HEK293T cells were transfected with HA-FSP1, Myc-ubiquitin, and Flag-tagged wild type TRIM21 (TRIM21-wt), or E3 ligase deficient TRIM21 (TRIM21-ELD), or binding domain deficient TRIM21 (TRIM21-BDD), or TRIM28. Whole cell lysates were analyzed by immunoblotting. D-E) HEK293T cells were transfected with HA-FSP1 and Myc-ubiquitin (wild type, K48O, K63O, K48R, K63R), with or without co-transfection of Flag-TRIM21. Whole cell lysates were analyzed by immunoblotting. F) HEK293T cells were transfected with Myc-ubiquitin (wild type, K6O, K11O, K27O, K29O, K33O),

with or without Flag-TRIM21. Cell lysates were analyzed by d-IP using anti-HA antibodies. G) HEK293T cells were transfected with Myc-Ubiquitin-K63O, full-length or truncated mutants of FSP1, with or without Flag-TRIM21. Whole cell lysates were analyzed by immunoblotting. H) HEK293T cells were transfected with Myc-Ubiquitin-K63O and wild type or five lysine (K) to arginine (R) mutants of FSP1. Whole cell lysates were analyzed by immunoblotting. I) HEK293T cells were transfected with Myc-Ubiquitin-K63O and wild type, K322R, K366R or K322R/K366R (2KR) mutant of FSP1. Whole cell lysates were analyzed by immunoblotting.

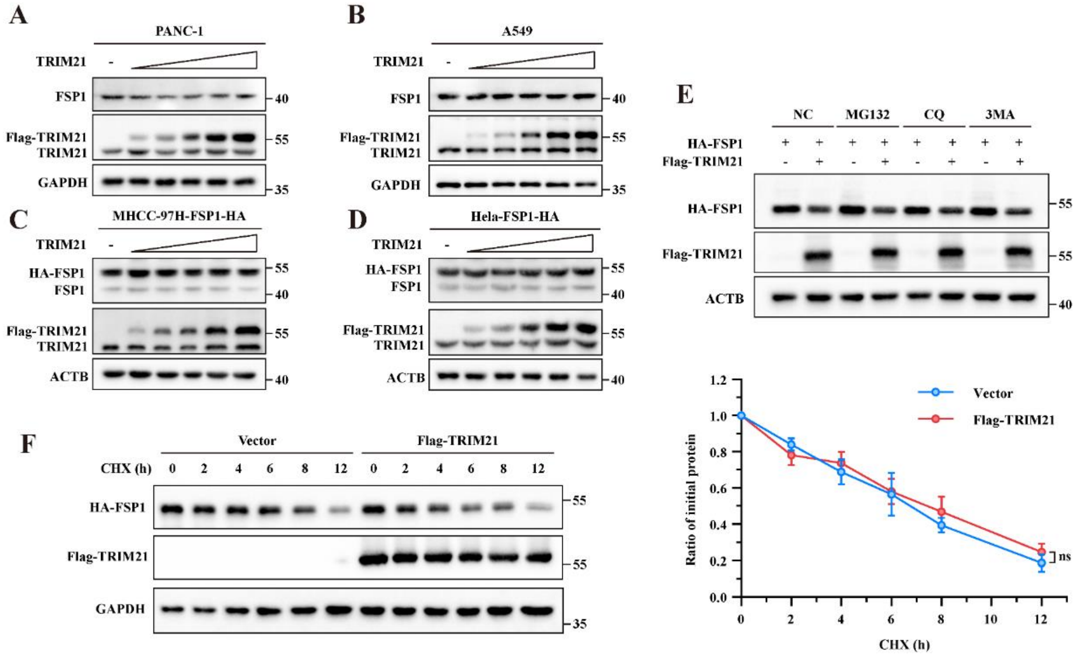

Figure S3. TRIM21 doesn't affect the protein level of FSP1. A-B) Expression level of FSP1 was determined by immunoblotting in PANC-1 (A) or A549 (B) cells transfected with increasing amounts of Flag-TRIM21. C-D) Expression level of FSP1 was determined by immunoblotting in MHCC-97H (C) or Hela (D) cells transfected with HA-FSP1 and increasing amount of Flag-TRIM21. E) MHCC-97H cells were transfected with HA-FSP1 with or without co-transfection of Flag-TRIM21 and treated with the proteasome inhibitor MG132 (10 μM, 6 h) or lysosome inhibitor CQ (20 μM, 6 h) or 3-MA (4 mM, 6 h). Expression level of FSP1 was determined by immunoblotting. F) MHCC-97H cells were transfected with HA-FSP1 and Flag-TRIM21 and treated with CHX (50 μM) for 0-12 h. Expression level of FSP1 was determined by immunoblotting. Representative images were shown in the left panel and quantitative analysis results were shown in the right panel ($n = 3$). Data were presented as means ± SD. P values were calculated by two-way ANOVA. ns, not significant.

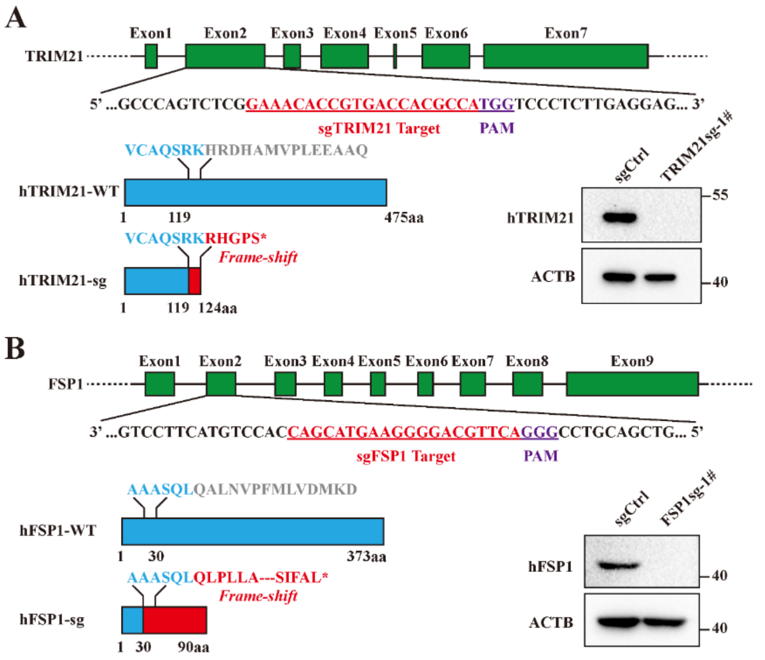

Figure S4. Construction of knockout cell lines using CRISPR/Cas9 technology. A) Schematic description of sgRNA target in the genome of human TRIM21 (upper panel) and amino acid sequence of isolated cell subclones verified by sanger sequencing (left panel). Immunoblotting was used to confirm the completely knockout of TRIM21 in the right panel. B) Schematic description of sgRNA target in the genome of human FSP1 (upper panel) and amino acid sequence of isolated cell subclones verified by sanger sequencing (left panel). Immunoblotting was used to confirm the completely knockout of FSP1 in the right panel.

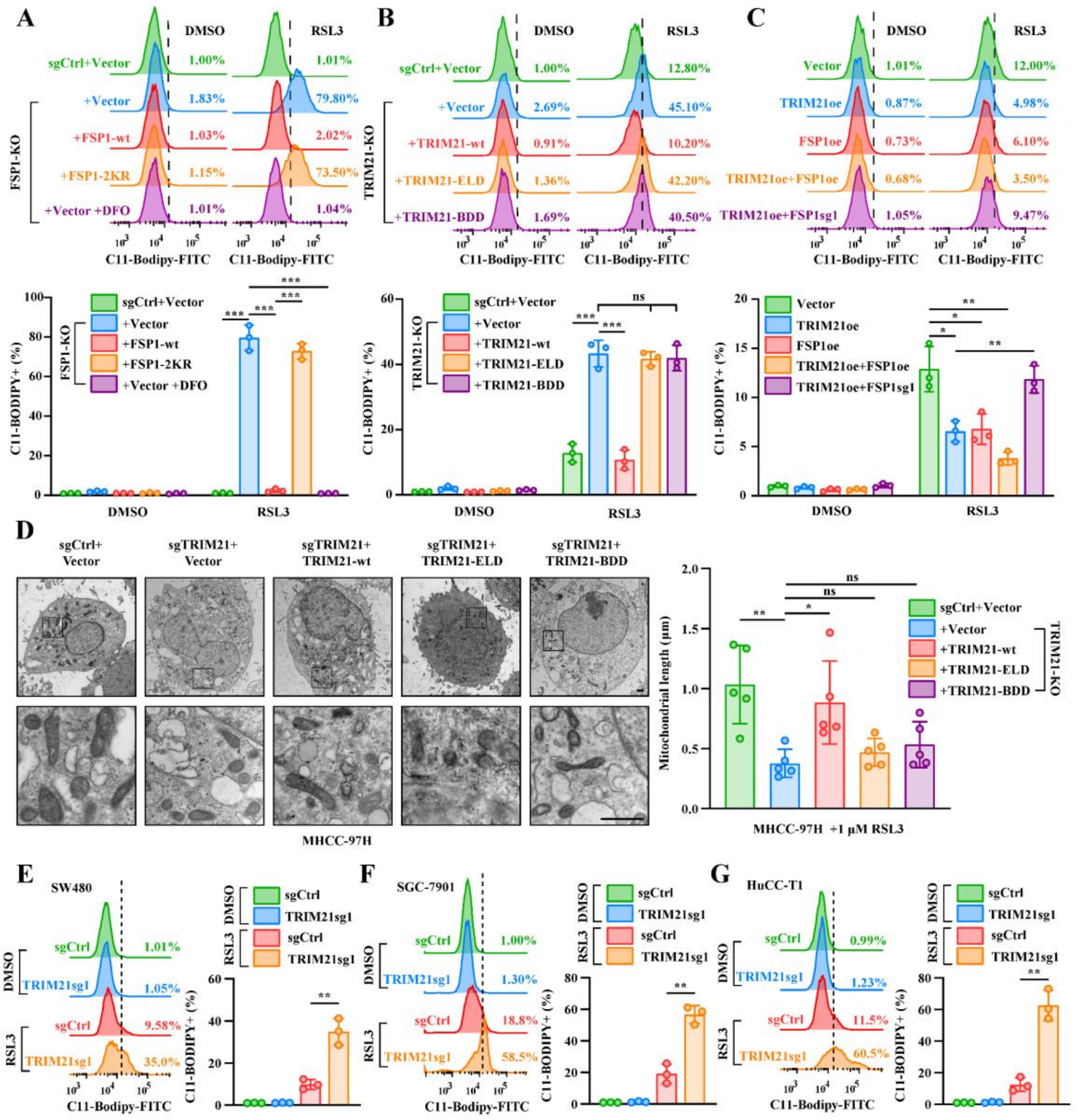

Figure S5. The protective role of TRIM21 against ferroptosis in GI tumor cells. A-C) KPC-1A cells transfected with indicated plasmids were treated with DMSO or 0.1 μM RSL3 for 1 h (A) or 1 μM RSL3 for 4 h (B and C) prior to C11-BODIPY staining. Flowcytometry were used to analyze the level of lipid peroxides as indicated by FITC fluorescence. Representative images of flowcytometry were shown in the upper panel. C11-BODIPY positive rate were counted in the lower panel (n = 3). Data were presented as means ± SD. D) MHCC-97H cells transfected with indicated plasmids were treated with 1 μM RSL3 for 6 h, and then were subjected to TEM analysis. Representative images were shown in the left panel. Mitochondrial shrinkage and mitochondrial ridge disappearance could be seen in cells with higher level of ferroptosis. Scale bars, 1 μm. Mitochondrial lengths along the long axis in each group were measured and summarized in the right panel. E-G) Cells were transfected with vector or sgTRIM21 plasmid and treated with DMSO or 1

μM RSL3 for 4 h prior to C11-BODIPY staining. Flowcytometry were used to analyze the level of lipid peroxides as indicated by FITC fluorescence. (E) SW480 cells; (F) SGC-7901 cells; (G) HuCC-T1 cells. Data were presented as means ± SD. *P* values were calculated by unpaired, two-tailed Student's *t*-test (A-D). *$P < 0.05$, **$P < 0.01$, ***$P < 0.001$, ns, not significant.

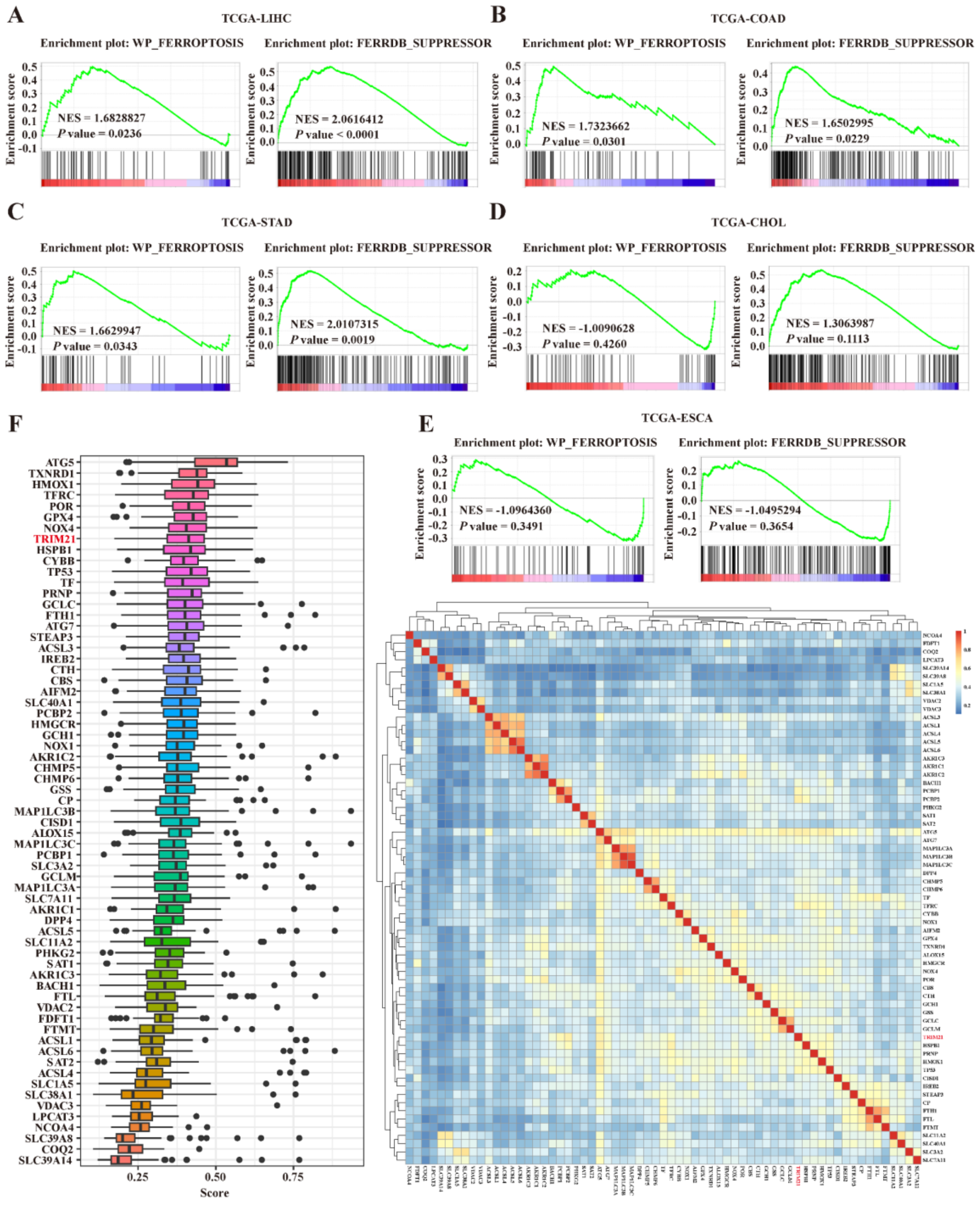

Figure S6. TRIM21 is associated with ferroptosis in multiple GI tumors. A-E) GSEA was conducted to explore the correlation between TRIM21 and ferroptosis-related genes based on LIHC (A), COAD (B), STAD (C), CHOL (D) and ESCA (E) dataset in TCGA database. F) Friends analysis was conducted to explore whether TRIM21 functions as an important hub gene in ferroptosis-related genes. Bar plot in the left panel shows the average score of each gene. Higher score indicates a closer relationship of the gene with other genes in the group based on GO annotation. Heatmap in the right panel shows the exact score of each gene with other genes in the group.

Supplemental Table 1. Key resources table

Reagent or resource	Source	Identifier
**Antibodies**		
MYC	Proteintech	Cat# 16286-1-AP
HA (For IB)	Proteintech	Cat# 51064-2-AP
HA (For IP)	Abclonal	Cat# AE008
FLAG (For IB)	Proteintech	Cat# 20543-1-AP
FLAG (For IP)	Sigma	Cat# F9291
GST	Abclonal	Cat# AE006
GAPDH	Proteintech	Cat# 60004-1-Ig
FSP1	Atlas	Cat# HPA042309
Caveolin-1	Proteintech	Cat# 16447-1-AP
Histone-H3	Proteintech	Cat# 17168-1-AP
COX-IV	Proteintech	Cat# 11242-1-AP
TRIM21	Proteintech	Cat# 12108-1-AP
ACTB	Abclonal	Cat# AC026
4-HNE	Bio-Techne	Cat# MAB3249
CK19	Proteintech	Cat# 10712-1-AP
α-SMA	Abcam	Cat# ab32575
Ki67	Abcam	Cat# ab15580
NRF2	CST	Cat# 12721
**Reagents**		
MG132	Selleck	Cat# S2619
RSL3	Selleck	Cat# S8155
IKE	Selleck	Cat# S8877
FIN56	Selleck	Cat# S8254
DFO	MCE	Cat# HY-B1625
DMSO	Sigma	Cat# 472301
Tween-80	MCE	Cat# HY-Y1891
PEG300	Selleck	Cat# S6704
Polyethyleneimine (PEI)	Sigma	Cat# 408727
Polybrene	Yeasen	Cat# 40804ES76
Protein A/G magnetic beads	Biolinkedin	Cat# L-1004A
Anti-Flag magnetic beads	Biolinkedin	Cat# L-1011A
MitoTracker Red CMXRos	ThermoFisher	Cat# M7512
DAPI	Servicebio	Cat# G1012
BODIPY™ 581/591 C11	ThermoFisher	Cat# D3861
DMEM	Cytiva	Cat# SH30022
RPMI-1640	Cytiva	Cat# SH30027
Cystine deprivation medium	Sangon biotech	N/A
**Critical Commercial Assays**		
Membrane and Cytosol Protein Extraction Kit	Beyotime	Cat# P0033
Nuclear and Cytoplasmic Protein Extraction Kit	Beyotime	Cat# P0028

Reagent or resource	Source	Identifier
Cell Mitochondria Isolation Kit	Beyotime	Cat# C3601
Cell Counting Kit-8	Dojindo	Cat# CK04
Cytotoxicity LDH Assay Kit	Dojindo	Cat# CK12
MDA assay kit	Beyotime	Cat# S0131
BCA assay kit	Beyotime	Cat# P0011
**Software and algorithms**		
Image Lab ™ Software 6.0	Bio-Rad	
GraphPad Prism 9.3.0	GraphPad	
FlowJo™ 10.4	FlowJo	
ZEN 3.6	Carl Zeiss	
GSEA 4.3.2	Broad Institute	
R 4.0.3	R Foundation	

Supplemental Table 2. Information for Primers used in this study

Oligos Sequences	
hTRIM21-shRNA#1	TGAGAAGTTGGAAGTGGAAAT
hTRIM21-shRNA#2	GAAGAGAGATTTGATAGTTAT
hTRIM21-sgRNA#1-F	CACCG GAAACACCGTGACCACGCCA
hTRIM21-sgRNA#1-R	AAAC TGGCGTGGTCACGGTGTTTCC
mTrim21-sgRNA#1-F	CACCG GAGCCTATGAGTATCGAATG
mTrim21-sgRNA#1-R	AAAC CATTCGATACTCATAGGCTCC
hFSP1-sgRNA#1-F	CACCG CAGCATGAAGGGGACGTTCA
hFSP1-sgRNA#1-R	AAAC TGAACGTCCCCTTCATGCTGC
mFsp1-sgRNA#1-F	CACCG CACGTGGTGATCGTGGGCGG
mFsp1-sgRNA#1-R	AAAC CCGCCCACGATCACCACGTGC
FSP1-pHAGE-F	TCGGGTTTAAACGGATCCGCCACC ATGGGGTCCCAGGTCTCGGTGGAAT
FSP1-pHAGE-R	GGGCCCTCTAGACTCGAGTCAAGGTGGAGACTGCCTCATGG
GST-FSP1-F	GGATCTGGTTCCGCGTGGATCC ATGGGGTCCCAGGTCTCGGTGGAAT
GST-FSP1-R	AGTCACGATGCGGCCGCTCGAG TCAAGGTGGAGACTGCCTCATGG
GST-TRIM21-F	GGATCTGGTTCCGCGTGGATCC ATGGCTTCAGCAGCACGCTTG
GST-TRIM21-R	AGTCACGATGCGGCCGCTCGAG ATAGTCAGTGGATCCTTGTGATCC
TRIM21-C16A-F	GGGAGGAGGTCACAGCCCCTATCTGCCTGGA
TRIM21-C16A-R	TCCAGGCAGATAGGGGCTGTGACCTCCTCCC
TRIM21-3133-F	CTGTGAGCATCGAGGCCGGCTGGAGCTTCTGCCAGGA
TRIM21-3133-R	TCCTGGCAGAAGCTCCAGCCGGCCTCGATGCTCACAG
TRIM21-W381383A-F	GGCTTCTGGACAATTGCCTTGGCCAACAAACAAAAATATGAG
TRIM21-W381383A-R	CTCATATTTTTGTTTGTTGGCCAAGGCAATTGTCCAGAAGCC
FSP1-K293R-F	CGTGAGGACGCCCAGAATGGCCTATCTTGCC
FSP1-K293R-R	GGCAAGATAGGCCATTCTGGGCGTCCTCACG
FSP1-K314R-F	CATCGTCAACTCTGTGAGACAGCGGCCTCTCC
FSP1-K314R-R	GGAGAGGCCGCTGTCTCACAGAGTTGACGATG
FSP1-K322R-F	CCTCTCCAGGCCTACAGACCGGGTGCACTGA
FSP1-K322R-R	TCAGTGCACCCGGTCTGTAGGCCTGGAGAGG
FSP1-K355R-F	TGGTTCGGCTGACCAGAAGCCGGGACCTG
FSP1-K355R-R	CAGGTCCCGGCTTCTGGTCAGCCGAACCA
FSP1-K366R-F	GTCTCTACGAGCTGGAGGACCATGAGGCAGTCT
FSP1-K366R-R	AGACTGCCTCATGGTCCTCCAGCTCGTAGAGAC

Supplemental Table 3. Gene sets used in GSEA analysis

Gene sets	Gene names
WP_ FERROPTOSIS	ACSL1 ACSL3 ACSL4 ACSL5 ACSL6 AIFM2 AKR1C1 AKR1C2 AKR1C3 ALOX15 ATG5 ATG7 BACH1 CBS CHMP5 CHMP6 CISD1 COQ2 CP CTH CYBB DPP4 FDFT1 FTH1 FTL FTMT GCH1 GCLC GCLM GPX4 GSS HMGCR HMOX1 HSPB1 IREB2 LPCAT3 MAP1LC3A MAP1LC3B MAP1LC3C NCOA4 NOX1 NOX4 PCBP1 PCBP2 PHKG2 POR PRNP SAT1 SAT2 SLC11A2 SLC1A5 SLC38A1 SLC39A14 SLC39A8 SLC3A2 SLC40A1 SLC7A11 STEAP3 TF TFRC TP53 TXNRD1 VDAC2 VDAC3
FERRDB_ SUPPRESSOR	SLC7A11 GPX4 AKR1C1 AKR1C2 AKR1C3 RB1 HSPB1 HSF1 GCLC NFE2L2 SQSTM1 NQO1 HMOX1 FTH1 MUC1 SLC3A2 MT1G SLC40A1 CISD1 FANCD2 FTMT HSPA5 ATF4 TP53 HELLS SCD FADS2 SRC STAT3 PML MTOR NFS1 TP63 CDKN1A MIR137 ENPP2 VDAC2 FH CISD2 CBS ISCU ACSL3 OTUB1 CD44 LINC00336 BRD4 PRDX6 SESN2 NF2 ARNTL HIF1A JUN CA9 TMBIM4 PLIN2 Fer1HCH AIFM2 LAMP2 ZFP36 PROM2 CHMP5 CHMP6 CAV1 GCH1 SIRT3 DAZAP1 PIR FTL HCAR1 SLC16A1 RRM2 NR4A1 PIK3CA RPTOR SREBF1 SREBF2 FZD7 P4HB NT5DC2 BCAT2 PLA2G6 PARK7 FXN SUV39H1 ATF2 ACOT1 ALDH3A2 STK11 FNDC5 CDH1 NEDD4L TF BRD2 BRD3 BRDT DECR1 GLRX5 NCOA3 NR5A2 PANX2 TFAP2A CP ARF6 GDF15 ABHD12 PPP1R13L TFAM KDM3B RNF113A AHCY circ-TTBK2 IDH2 PPARA NOS2 SIAH2 RELA PRKAA2 VDR NEDD4 PRDX1 AR MTF1 COPZ1 NUPR1 USP35 PARP1 PARP2 PARP3 PARP4 PARP6 PARP8 PARP9 PARP10 PARP11 PARP12 PARP14 PARP15 PARP16 PDSS2 TXN SENP1 FGF21 CREB1 CREB3 CREB5 GOT1 TFRC BEX1 ASAH2 FABP4 AKT1S1 MLST8 SIRT1 TYRO3 SIRT6 TMSB4X TMSB4Y KIF20A ECH1 ETV4 VCP RBMS1 KDM4A MGST1 MPC1 CHMP1A CAMKK2 SOX2 SRSF9 PROK2 SIRT2 MEF2C EZH2 PEDS1 SMPD1 ADAMTS13 CDC25A G6PD PPARD ENO3 LCN2 MARCHF5 TRIB2 DHODH PDK4 ADIPOQ IL6 PTPN18 ABCC5 CISD3 MS4A15 FURIN GALNT14 KLHDC3 MAPKAP1 PRR5 RICTOR GSTM1 TERT RARRES2 USP11
